# The Effect of Photoisomerization on the Antioxidant Properties of Sinapic Acid and Methyl Sinapate in Different Solvents: A DFT/TD-DFT Study

**DOI:** 10.3390/antiox14060633

**Published:** 2025-05-25

**Authors:** Lei Wang, Chaofan Sun, Lingling Wang

**Affiliations:** 1 College of Science, Northeast Forestry University, Harbin 150040, China; wangl@nefu.edu.cn; 2 College of Chemistry, Chemical Engineering and Resource Utilization, Northeast Forestry University, Harbin 150040, China; 3 Key Laboratory of Forest Plant Ecology, Ministry of Education, Northeast Forestry University, Harbin 150040, China; 4 Heilongjiang Provincial Key Laboratory of Ecological Utilization of Forestry-Based Active Substances, Northeast Forestry University, Harbin 150040, China

**Keywords:** sinapic acid, photoisomerization, antioxidant, dual descriptor potential, density function theory

## Abstract

The impact of photoisomerization on antioxidant properties holds significant implications for fields such as medicine, chemistry, and consumer products. This investigation employs multistate complete active space second-order perturbation theory (MS-CASPT2), complemented by density functional theory (DFT) and time-dependent DFT (TD-DFT) methods, to examine the photoisomerization behavior of sinapic acid (SA) and methyl sinapate (MS) under ultraviolet (UV) irradiation, while systematically analyzing their antioxidant properties in the S_1_ state. The computational results, validated by two independent theoretical approaches, confirm that both SA and MS can undergo photoisomerization through conical intersection pathways, providing crucial insights into their non-radiative transition mechanisms. In the S_0_ state, cis-SA and cis-MS exhibit higher antioxidant activity, while in the S_1_ state, antioxidant performance is strongly solvent-dependent: trans-SA outperforms in ethyl acetate (Eac) and water, whereas cis-SA is more effective in methanol (MeOH). Notably, the natural population analysis (NPA) charges of all four compounds increase upon photoexcitation, suggesting that photoexcitation enhances antioxidant properties. This study addresses a critical gap in our understanding of the relationship between photoisomerization and antioxidant activity in natural phenolic compounds.

## 1. Introduction

Antioxidants play a pivotal role in mitigating oxidative stress, which is implicated in various diseases and aging processes [1,2,3]. Their applications span diverse fields, including medicine, food preservation, and cosmetics, driving extensive research into understanding and optimizing their activity [4,5,6]. Among natural antioxidants, phenolic acids such as sinapic acid (SA) and methyl sinapate (MS) have attracted significant attention due to their potent radical-scavenging capability and structural versatility via photoisomerization [7,8,9].

Photoisomerization, a light-induced structural transformation, is a key phenomenon in photochemistry that can alter the electronic and geometric properties of compounds, thereby influencing their chemical characteristics and biological activity [10,11,12]. For instance, studies indicate that the antibacterial activity of pyridine derivatives depends on their geometric configuration, with cis- and trans-isomers exhibiting varied effects on different bacterial strains [13]. In 2007, 2,2-Diphenyl-1-picrylhydrazyl (DPPH) radical scavenging and lipid peroxidation experiments demonstrated superior antioxidant activity in cis-astaxanthin compared to its all-trans form [14]. More recently, novel spectroscopic techniques combined with singlet oxygen quenching assays reaffirmed the enhanced antioxidant efficacy of cis-isomers over trans-isomers [15]. In 2024, Alejandra and collaborators isolated trans- and cis-p-coumaroyl-secologanosides from olive oil waste, showing through DPPH and 2,2′-azino-bis(3-ethylbenzothiazoline-6-sulfonic acid) (ABTS^•+^) radical-scavenging assays that trans-isomers possess stronger radical-scavenging capabilities than their cis counterparts [16]. In addition to photoisomerization, solvent polarity also has a profound impact on the antioxidant properties of compounds. Higher solvent polarity leads to a lower thermal isomerization barrier for the cis-to-trans transition of 4-aminoazobenzene, resulting in an increased reaction rate [17]. Previous studies have primarily focused on the S_0_-state properties of antioxidants, while investigations into their S_1_-state behavior and environmental dependence remain limited. This knowledge gap hinders the rational design of light-responsive antioxidants with tunable activity.

**Figure 1:**
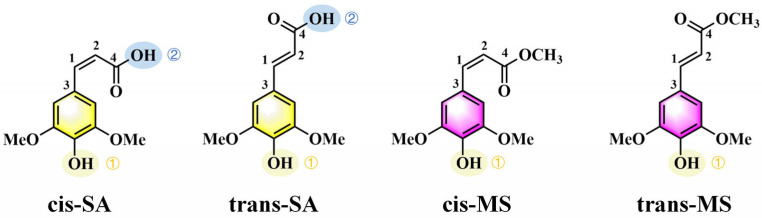
Chemical structures of cis-SA, trans-SA, cis-MS, and trans-MS.

In this study, we aim to bridge this gap by elucidating the effects of photoisomerization and solvent polarity on the antioxidant activity of SA and MS. The molecular structures of SA and MS are presented in Figure 1. Density functional theory (DFT) and time-dependent DFT (TD-DFT) were employed to evaluate antioxidant performance based on various parameters. Based on the linearly interpolated internal coordinate (LIIC) method, we constructed the photoisomerization potential energy curves of the target compounds, demonstrating their energy relaxation through the photoisomerization process. The simulated absorption spectra demonstrate that these compounds exhibit strong ultraviolet (UV) absorption capabilities in both the UVC and UVA regions. Furthermore, analyses of their frontier molecular orbitals (FMOs) and global descriptive parameters provided deeper insights into the influence of cis/trans isomerization and solvent effects on antioxidant performance. Additionally, by examining molecular electrostatic potential (MEP), natural population analysis (NPA) charges, and dual descriptor potential, we identified the radical reaction sites and compared the radical-scavenging capabilities in both the S_0_ and S_1_ states. These findings provide valuable insights into the role of photoisomerization and solvent effects in modulating the antioxidant activity of phenolic compounds, paving the way for the development of novel antioxidant compounds.

## 2. Materials and Methods

The molecular geometries of SA and MS were determined using DFT [18,19] and TD-DFT [20,21,22] methods optimized at the B3LYP/6-311G(d,p) [19,23] computational level. This combination provided a robust framework for describing both S_0_ and S_1_ electronic states with reasonable computational cost. The B3LYP functional was selected due to its demonstrated reliability in predicting the structures and properties of organic phenolic compounds with aromatic and conjugated systems similar to our target molecules.

Conical intersection (CI) geometries, which play crucial roles in photoisomerization pathways, were precisely located using the Restricted Active Space Self-Consistent-Field (RASSCF) [24,25] method implemented in OpenMolcas v24.06 [26,27]. The RASSCF method offers significant advantages over conventional CASSCF [28,29] calculations in CI optimizations [30,31]. First, it provides higher efficiency and greater flexibility. The active space in the RASSCF calculation is divided into three distinct subspaces: RAS1, RAS2, and RAS3. RAS1 allows a limited number of electrons to be moved from occupied orbitals, while RAS3 allows a limited number of electrons to enter unoccupied orbitals [32]. This partitioning reduces computational costs while retaining essential electronic correlations, making it particularly suitable for CI optimizations in complex molecular systems [32]. For the SA molecular framework, we implemented an active space comprising 2 electrons distributed across 3 orbitals (denoted as (2,3)). For MS, the active space consisted of 4 electrons distributed across 4 orbitals (4,4). These active spaces incorporated the critical π/π* orbitals that characterize the conjugated electronic system, which was essential for the accurately modeling of photochemical properties. Orbital visualizations and comprehensive justification for our active space selection methodology are presented in Figures S1 and S2 of the Supplementary Materials. We implemented a state-averaging procedure across four electronic states to maintain quantum mechanical consistency between states and mitigate potential root-flipping complications during geometric optimization calculations.

The potential energy surfaces of photoisomerization were accurately calculated using the multistate complete active space second-order perturbation theory (MS-CASPT2) [33,34] method in combination with the def2-TZVP basis set, revealing the relaxation pathways. MS-CASPT2 is one of the most successful quantum chemistry methods in photochemistry for capturing both static and dynamic electron correlation effects [35], which are essential for describing S_1_-state processes. MS-CASPT2 incorporates second-order perturbation theory to correct for dynamic correlation effects, significantly improving energy predictions [36]. Moreover, this method supports the PCM solvent model, providing a reliable theoretical framework for investigating the influence of solvent effects on the photoisomerization process. To balance computational cost and accuracy, an imaginary level shift of 0.2 atomic units was also implemented to address potential intruder-state problems in perturbation calculations. Further, we performed a constrained potential energy surface scan at the CAM-B3LYP/6-311+G(d,p) level using the TD-DFT method. This method allowed for geometric relaxation along each fixed dihedral angle in the isomerization coordinates.

FMOs, MEP, and dual descriptor potential were visualized using Multiwfn 3.8 [37,38] in conjunction with VMD 1.9.4 [39]. These computational tools allowed for the detailed investigation of electronic structure and reactivity features across different molecular states and environments. Global descriptive parameters, which provide valuable insights into molecular reactivity patterns, were calculated according to conceptual DFT. These parameters, which included ionization potential (IP), electron affinity (EA), hardness (η), softness (S), chemical potential (μ), electrophilicity (ω), and electronegativity (χ), were calculated using the following formulas [40,41,42,43].

IP = E(N − 1) − E(N)(1)

EA = E(N) − E(N + 1)(2)

hardness (η) = (IP − EA)/2(3)

softness (S) = 1/2η(4)

electronegativity (χ) = (IP + EA)/2(5)

chemical potential (μ) = −electronegativity (χ)(6)

electrophilicity (ω) = μ^2^/2 (7)

Fukui functions (f^−^, f^+^, and f^0^) are widely used to predict reactive sites but are only indirectly related to the external potential at the initial stage of the reaction. To overcome this limitation, we implemented the more recently developed Fukui potential V_f−/+/0_(r), which provides a more direct description of reactivity by incorporating spatial information. The difference between f^−^ and f^+^ yields the dual descriptor (∆f), which highlights nucleophilic and electrophilic regions. Recently, the dual descriptor potential (DDP), obtained by performing a Coulomb integral on Δf, has been proposed. It offers theoretical advantages and improved accuracy in predicting reactive sites compared to the dual descriptor [44]. All DFT/TD-DFT calculations in this work were performed using Gaussian 16 [45].

$$V_{f^{-/+/0}}(r)=\int \frac{f^{-/+/0}(r^{\prime })}{|r-r\prime |}dr\prime$$

$$DDP\left(r\right)=\int \frac{∆f\left(r^{\prime }\right)}{|r-r^{\prime }|}dr^{\prime }=\int \frac{f^{+}\left(r^{\prime }\right)-f^{-}\left(r^{\prime }\right)}{|r-r^{\prime }|}dr^{\prime }=V_{f^{+}}(r)-V_{f^{-}}(r)$$

## 3. Results and Discussion

### 3.1. Spectral Properties and Frontier Molecular Orbitals (FMOs)

To better understand the photophysical behavior of the studied compounds, we conducted a detailed analysis of their geometric structures and electronic properties in different solvents. Table 1 presents the dihedral angles D_3-1-2-4_ and D_1-2-4-o_ for cis- and trans-isomers of SA and MS, in both the S_0_ and S_1_ states, across three solvents: ethyl acetate (Eac), methanol (MeOH), and water. The Cartesian coordinates for both the S_0_ and S_1_ states are comprehensively documented in Tables S1–S6 of the Supplementary Materials. These angles are essential indicators of the degree of planarity or twisting within the molecular framework, which in turn affects the π-conjugation and overall electronic delocalization. In general, dihedral angles approaching 0° or 180° indicate more planar conformations, conducive to enhanced π-orbital overlap. For instance, the trans-isomers of both SA and MS consistently exhibit dihedral angles close to ±180°, indicating an extended planar configuration that facilitates conjugation across the molecule. On the other hand, cis-isomers, especially cis-MS, show significant deviations from planarity in more polar solvents. For instance, in water, cis-MS exhibits D_1-2-4-o_ angles of ~18.9° in the S_0_ state, indicating a pronounced twist that may influence its absorption behavior.

**Figure 2:**
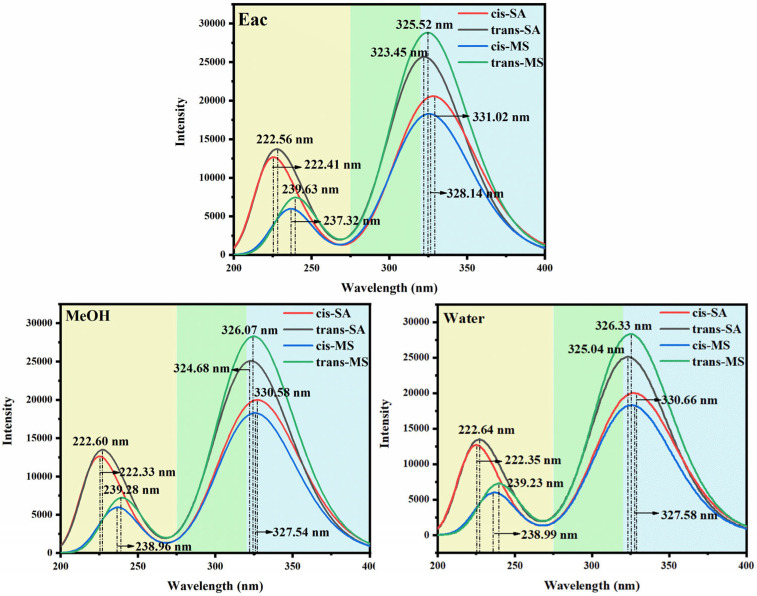
Simulated absorption of cis–trans-isomers of SA and MS.

Figure 2 and Table S7 present the UV-Vis absorption spectra and transition properties calculated at the B3PW91/6-311++G(d,p) level of theory using implicit solvent models. All the compounds exhibit absorption in both the UVC (<280 nm) and UVA (320–400 nm) regions, with more intense peaks generally observed in the UVA range. This is significant from a photobiological perspective, as UVA radiation is particularly harmful due to its deep penetration into the dermis, where it can generate reactive oxygen species, leading to premature skin aging and increased cancer risk [46,47,48]. The pronounced UVA absorption exhibited by trans-SA and trans-MS (as demonstrated by the black and green curves in Figure 2) highlights their potential as effective photoprotective agents. In the Eac solution, for example, trans-SA and trans-MS exhibit oscillator strengths of 0.6059 and 0.6883, respectively, substantially higher than those of cis-SA and cis-MS (0.4490 and 0.4336). In contrast, in the UVC region, a different trend emerges: SA isomers (both cis and trans) display stronger absorptions than their corresponding MS isomers. Table S7 shows that in Eac, the oscillator strengths of cis-SA and trans-SA in the UVC region are 0.2550 and 0.2464, respectively, while those of cis-MS and trans-MS are merely 0.0659 and 0.1195. This spectral difference can be attributed to the disparate electronic effects between the carboxylic acid group (-COOH) and the methyl ester moiety (-COOCH_3_).

**Figure 3:**
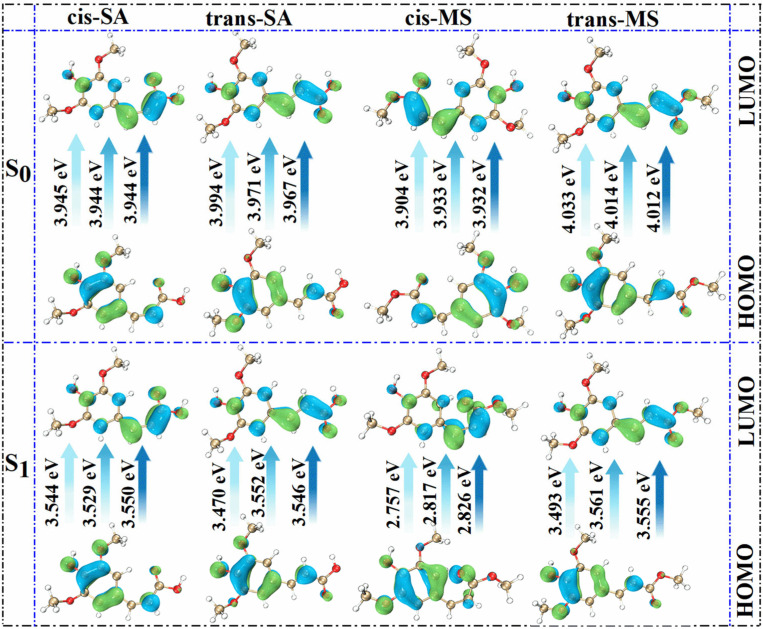
Molecular orbital diagrams of investigated compounds in three solvents in S_0_ and S_1_ states.

To further rationalize the spectral behavior, FMO diagrams were analyzed (Figure 3). These diagrams illustrate the energy gaps between the highest occupied molecular orbital (HOMO) and the lowest unoccupied molecular orbital (LUMO), which primarily govern electronic transitions from the S_0_ state to the S_1_ state. The color-coded arrows represent solvent polarity, ranging from low (Eac) to high (water). A reduced HOMO–LUMO gap corresponds to a lower excitation energy and thus a longer wavelength for the absorption maximum [49,50,51,52]. The data reveal that cis-isomers generally possess smaller HOMO-LUMO gaps, consistent with their red-shifted absorption wavelengths. In Eac solutions, cis-SA exhibits a gap of 3.945 eV, smaller than that of trans-SA at 3.994 eV; similarly, cis-MS shows a gap of 3.904 eV, smaller than that of trans-MS at 4.033 eV. This energy gap differentiation perfectly explains the observed absorption wavelength trends: 331.02 nm (cis-SA) > 323.45 nm (trans-SA) and 328.14 nm (cis-MS) > 325.52 nm (trans-MS). According to Table 1, cis-MS undergoes significant torsional changes in the S_1_ state, especially in the D_3-1-2-4_ angle, which increases to over 10° in all solvents. This suggests the less favorable overlap of π orbitals in the S_1_ state, potentially altering oscillator strengths and shifting absorption behavior.

In conclusion, the UV-Vis absorption characteristics of SA and MS are jointly influenced by molecular conformation and isomerism. The disparities between SA and MS series stem from the different electronic effects of their carboxylic acid groups and methyl ester groups, while the spectral differences between cis- and trans-isomers can be attributed to variations in conformational planarity and HOMO-LUMO energy gaps. These findings not only shed light on fundamental structure–property relationships but also support the potential use of these compounds in UV-blocking or photo-responsive applications.

### 3.2. Photoisomerization Process of Two Compounds

**Figure 4:**
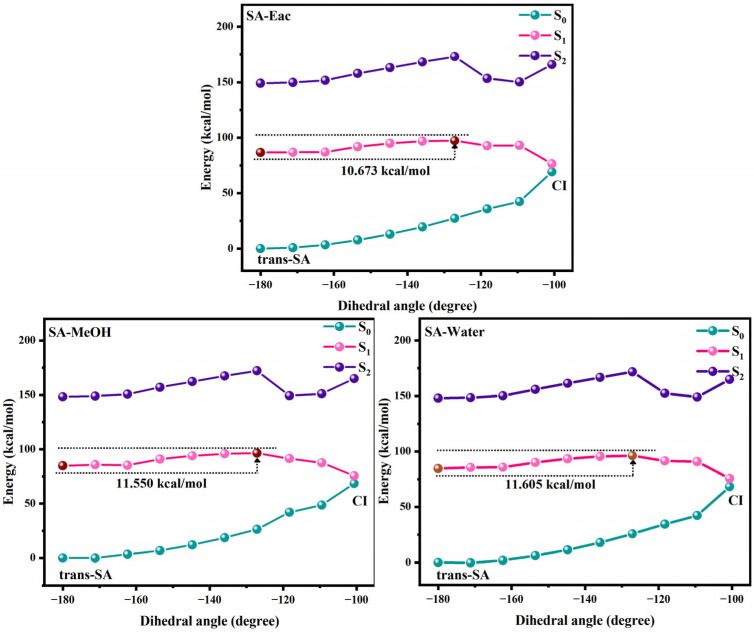
The photoisomerization relaxation processes of SA.

**Figure 5:**
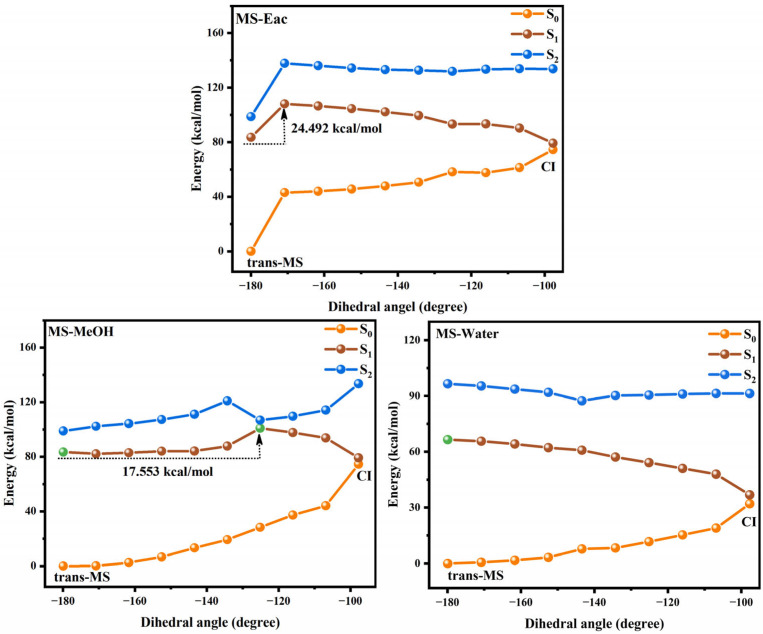
The photoisomerization relaxation processes of MS.

Figure 4 and Figure 5 show the potential energy curves for the photoisomerization of SA and MS in three solutions, constructed using the LIIC method. The LIIC method, widely applied in the literature, reliably describes potential energy surfaces and yields consistent results [8,53,54,55]. Figures S3 and S4 demonstrate the relaxation scans performed for C_3_-C_1_=C_2_-C_4_ at the CAM-B3LYP/6-311+G(d,p) level based on the TD-DFT method. Photoisomerization refers to a photochemical reaction in which a compound undergoes structural rearrangement after absorbing photons under photoexcitation [56,57]. In the S_1_ state, the compound undergoes nuclear motion to lower its potential energy, ultimately undergoing a non-radiative transition at a CI [58,59]. During this process, the compound may relax to either the trans or cis structure, indicating that the cis and trans configurations of SA and MS coexist under standard conditions in dynamic equilibrium. This coexistence provides strong evidence supporting the investigation of the impact of cis–trans isomerization on antioxidant properties. Furthermore, using the RASSCF [30,60,61] method in OpenMolcas, the CI of SA and MS in MeOH solution was accurately located. The dihedral angles of C_3_-C_1_=C_2_-C_4_ were found to be 100.7° and −97.7°, respectively. The Cartesian coordinates of their molecular structures are provided in the Supplementary Materials (Tables S8–S13). These results are in excellent agreement with the value of −94° obtained from Firefly calculations for the CI in an MeOH solution, further validating the reliability of our results [8]. Subsequently, we determined the CI angles of SA and MS in three different solvents, observing a dihedral angle of −100.7° for SA in both Eac and water, while MS displayed an angle of −98.1°.

We investigated the photoisomerization process of SA and MS using two distinct computational approaches: the MS-CASPT2 method combined with LIIC, and the TD-DFT method with constrained potential energy surface scanning. These methodologies exhibit notable differences in their prediction of photoisomerization energy barriers. The TD-DFT calculations reveal relatively modest energy barriers for SA in Eac, MeOH, and water (2.970, 3.263, and 3.256 kcal/mol, respectively), with comparable values for MS across these solvents (3.427, 3.412, and 3.403 kcal/mol, respectively). In contrast, the MS-CASPT2 approach predicts substantially higher barriers: 10.673, 11.550, and 11.605 kcal/mol for SA, and 24.492, 17.553 kcal/mol, and no barrier for MS in the respective solvents. This discrepancy primarily stems from the fundamental theoretical differences between the two methods. TD-DFT, as a single-reference approach, has inherent limitations in treating excited states with significant multi-configurational characteristics, whereas MS-CASPT2, as a multi-reference method, more comprehensively accounts for electron correlation effects. Nevertheless, TD-DFT offers the advantage of enabling a more complete scan of the isomerization pathway. Despite their quantitative differences, both methodologies converge on key qualitative insights regarding the photoisomerization mechanism. The calculations collectively demonstrate that SA and MS undergo photoisomerization through pathways mediated by conical intersections, which function as essential conduits for non-radiative electronic transitions between the S_1_ and S_0_ states during the isomerization process. Based on these results, we suggest that the changes in the molecular conformations of SA and MS by the photoisomerization process will have an important impact on their antioxidant properties.

### 3.3. Active Site

**Figure 6:**
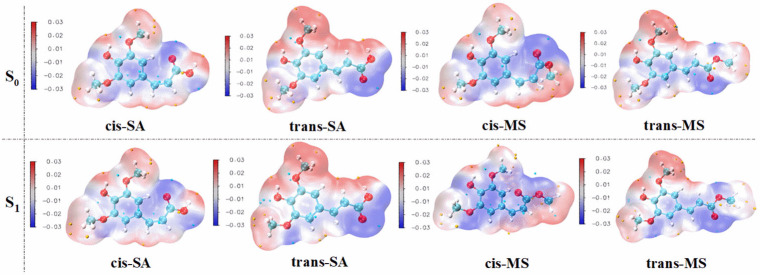
The distribution of the electrostatic potential of the compounds in their S_0_ and S_1_ states. (Blue regions indicate electron-rich areas, while red regions indicate electron-deficient areas.).

Molecular electrostatic potential (MEP) serves as a valuable tool for visualizing the electrostatic potential distribution around a compound, revealing long-range interactions and identifying nucleophilic and electrophilic reactive sites [62]. Figure 6 presents the MEP maps of cis-SA, trans-SA, cis-MS, and trans-MS in their S_0_ states and S_1_ states within the Eac solvent. Additional MEP maps in MeOH and water are provided in Figures S5 and S6 of the Supplementary Materials. The MEP analysis reveals that the aromatic ring and its hydroxyl oxygen appear as blue regions, indicating high electron density and a strong affinity for radicals. These electron-rich areas likely serve as primary sites for the hydrogen atom transfer or single-electron transfer processes that characterize antioxidant activity. However, it is important to acknowledge that MEP has inherent limitations in accurately describing covalent bond formation dynamics, which consequently restricts its ability to precisely pinpoint reactive sites involved in antioxidant mechanisms. This limitation arises because MEP primarily focuses on electrostatic interactions rather than the quantum mechanical effects associated with bond breaking and formation.

**Figure 7:**
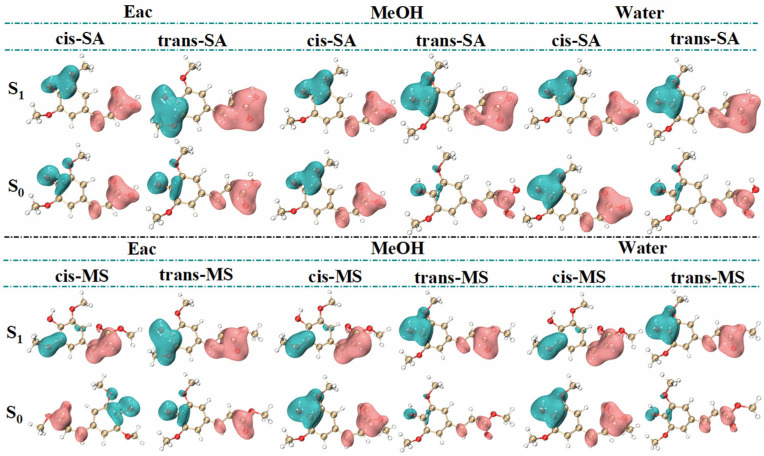
DDP images of several compounds in S_0_ and S_1_ states. (Green regions (negative values) represent electrophilic sites, while pink regions (positive values) represent nucleophilic sites.).

To address these constraints and gain more comprehensive insights, dual descriptor potential (DDP) has been introduced as an advanced reactivity descriptor directly correlated with energy [44]. DDP offers several advantages over traditional reactivity indices, including biphasic properties and high-resolution 3D imaging that enable the clear identification of nucleophilic and electrophilic regions. Unlike simpler descriptors, based solely on charge distribution, DDP incorporates both electron donation and acceptance capabilities, providing a more nuanced picture of molecular reactivity across various environments. Figure 7 reveals consistent molecular distribution patterns across solvents and shows significantly enlarged isosurfaces in the S_1_ state, indicating enhanced reactivity. In SA, the hydroxyl group on the benzene ring emerges as the most prominent electrophilic site, while the carboxyl groups and hydrogen atoms in the C=C bond serve as the main nucleophilic sites. Similarly, in MS, the electrophilic site is near the hydroxyl group, while the nucleophilic sites are concentrated around the carbon–carbon double bond, its connected ester group, and the hydrogen atoms. Notably, in the S_1_ state, the electrophilic site of cis-MS shifts from the hydroxyl group to the oxygen atom of the methoxy group attached to the benzene ring.

To quantitatively investigate reactive sites beyond this visual analysis, NPA charges were calculated [41,63]. The results, as shown in Table 2, indicate that H_1_ in cis-SA and trans-SA carries a higher positive charge than H_2_, indicating H_1_ as the preferred hydrogen-donating site for radical-scavenging reactions. Furthermore, a comparison of charge distribution across the four compounds in different electronic states reveals that the charges in the S_1_ state are significantly higher than in the S_0_ state, further supporting the notion that the S_1_ state exhibits greater chemical reactivity. For example, the charge distributions of cis-SA in the three solvents are as follows: in Eac, 0.49140 (S_0_) < 0.49708 (S_1_); in MeOH, 0.49371 (S_0_) < 0.50263 (S_1_); and in water, 0.49405 (S_0_) < 0.50607 (S_1_). This increased polarization in the S_1_ state could facilitate hydrogen abstraction, potentially enhancing antioxidant capacity under photoexcitation conditions. Additionally, it was observed that the charge on the reactive sites significantly increases with solvent polarity. Taking the H_1_ site of cis-SA as an example, the charge in the S_0_ state follows the following trend: 0.49149 (Eac) < 0.49371 (MeOH) < 0.49405 (water). Similarly, in the S_1_ state, the charge increases as follows: 0.49708 (Eac) < 0.50263 (MeOH) < 0.50607 (water). These results indicate that higher solvent polarity enhances the chemical reactivity of the compounds, potentially leading to improved antioxidant properties in polar environments such as cellular aqueous media.

**Figure 8:**
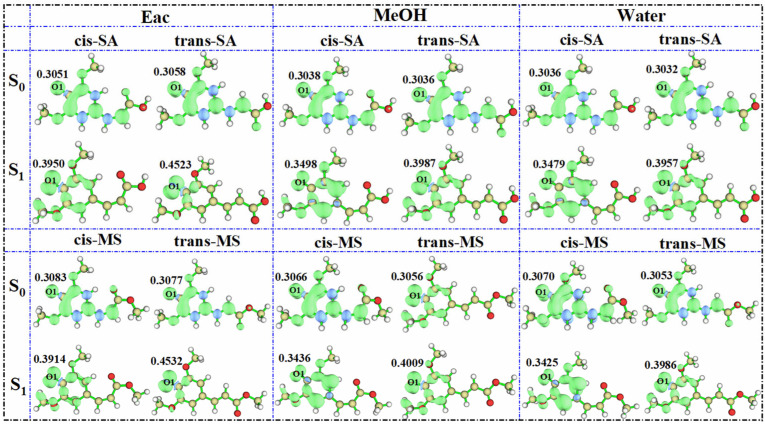
Spin densities of SA and MS radicals in three solvents in the S_0_ and S_1_ states.

To evaluate the antioxidant potential more comprehensively, the most probable hydrogen-donating sites were identified based on NPA and the hydroxyl hydrogen on O_1_ being the most electrophilic. Subsequently, we simulated the spin density distribution of the resulting radical species after H-abstraction at this site. This approach provides a useful proxy for assessing the stability of radical intermediates, which is known to correlate with antioxidant effectiveness [64,65]. More stable radicals typically indicate more efficient antioxidant activity, as they prevent further oxidative chain reactions. The results (Figure 8) show that as solvent polarity increases, the spin density of the investigated compounds decreases. For cis-SA in the S_0_ state, the spin density values follow the following order: 0.3051 (Eac) > 0.3038 (MeOH) > 0.3036 (water). This inverse relationship between solvent polarity and spin density indicates that radicals generated in high-polarity solvents exhibit enhanced delocalization and greater stability, thereby potentially improving antioxidant properties. The observed trend aligns with the general principle that greater spin delocalization correlates with improved radical-scavenging capacity, as it increases the kinetic stability of the radical intermediates. Interestingly, the spin density of the oxygen atoms in S_1_-state radicals shows a contrary trend, increasing rather than decreasing. This phenomenon can be attributed to photoexcitation increasing the overall reactivity of the molecule, making it more active when it becomes a radical. The heightened reactivity in the S_1_ state could potentially enable these compounds to neutralize more reactive radical species that might be resistant to S_0_-state antioxidants, suggesting unique applications in photodynamic therapy or photoprotection.

### 3.4. Global Descriptive Parameters of cis–trans-SA and -MS in Different Solvents

Global descriptive parameters are commonly used to assess molecular reactivity and elucidate molecular behavior in various chemical environments, including ionization potential (IP), electron affinity (EA), hardness (η), softness (S), chemical potential (µ), electrophilicity (ω), and electronegativity (χ). Together, these parameters provide a comprehensive picture of molecular capacity for electron transfer, which is crucial in processes such as antioxidant activity, where electron donation and acceptance play central roles. In this study, descriptive factors for four geometric isomers—cis-SA, trans-SA, cis-MS, and trans-MS—were calculated and analyzed in three solvents—Eac, MeOH, and water—representing a gradient of increasing polarity. This approach enabled the systematic evaluation of solvent effects on molecular properties, with the results being summarized in Table 3.

IP represents the energy required for an electron to escape from a molecule; thus, a lower IP indicates higher electron donation ability and better molecular reactivity [66,67]. This property is crucial in radical-scavenging reactions, where molecules need to donate electrons to neutralize harmful radicals. Chemical hardness indicates a molecule’s resistance to electron cloud deformation and polarization in chemical processes; lower hardness implies higher reactivity. Comparing S_0_- and S_1_-state IP values across the four molecules reveals significant changes. For example, cis-SA exhibits values of 5.9671 eV (S_0_) > 5.8363 eV (S_1_) in Eac, 5.7512 eV (S_0_) > 5.6010 eV (S_1_) in MeOH, and 5.7225 eV (S_0_) > 5.5516 eV (S_1_) in water. Evidently, IP values decrease markedly upon photoexcitation, enhancing the electron-donating capacity to free radicals and consequently improving antioxidant activity. In addition, the data further demonstrate an inverse correlation between solvent polarity and IP values, as exemplified by cis-MS in both electronic states: 5.9671 eV (Eac) > 5.7512 eV (MeOH) > 5.7225 eV (water) and 5.1760 eV (Eac) > 5.0366 eV (MeOH) > 5.0200 eV (water). This progressive decrease in IP with increasing solvent polarity indicates enhanced molecular reactivity and, consequently, improved antioxidant capacity in more polar environments. The observed trend is consistent across all studied isomers, suggesting that polar solvents facilitate electron donation by stabilizing the resulting cationic species through solvation effects. Chemical hardness also exhibits significant changes with photoexcitation and varying solvent polarity, as exemplified by trans-SA parameters: 4.5056 eV (Eac) > 3.9729 eV (MeOH) > 3.9007 eV (water) in S_0_ and 3.9837 eV (Eac) > 3.5235 eV (MeOH) > 3.4489 eV (water) in S_1_. Notably, trans-SA in the S_1_ state exhibited the lowest chemical hardness of 3.4489 eV in water, further confirming that both photoexcitation and increased solvent polarity enhance antioxidant properties.

There is also a trend that the further analysis of the impact of cis–trans isomerization on antioxidant properties reveals distinct trends. In the S_0_ state, cis-SA and cis-MS exhibit lower chemical hardnesses than their trans counterparts, indicating better antioxidant activity. However, the situation becomes more complex in the S_1_ state. In Eac and water, trans-SA has the lower chemical hardness, while in MeOH, cis-SA is more favorable. For instance, in Eac, the chemical hardness of trans-SA (3.9837 eV) is lower than that of cis-SA (4.0338 eV), whereas in MeOH, cis-SA (3.4987 eV) outperforms trans-SA (3.5235 eV). In water, trans-SA (3.4489 eV) also shows slightly lower hardness than cis-SA (3.4518 eV). Notably, cis-MS consistently shows superior chemical properties across all solvents.

## 4. Conclusions

Through comprehensive quantum chemical calculations, we elucidated the intricate relationship between photoisomerization processes, solvent polarity effects, and the resultant antioxidant efficacy of SA and MS. The computational analysis of absorption spectra demonstrated that both compounds exhibited significant absorption capabilities in the UVA (320–400 nm) and UVC (100–280 nm) regions, with the subsequent dissipation of excitation energy occurring predominantly via CI. We employed complementary computational approaches to characterize the photoisomerization mechanism, including LIIC path mapping and constrained potential energy surface scanning with TD-DFT. These calculations confirmed the existence of energy-accessible photoisomerization pathways via conical intersections, providing crucial insights into the non-radiative transition mechanisms of cis–trans interconversion upon photoexcitation. NPA and DDP analyses further substantiate that UV irradiation significantly amplifies the antioxidant capacities of these compounds and identify H_1_ as the preferred reactive site for radical scavenging. Additionally, the analysis of global descriptors reveals that increasing solvent polarity significantly enhances molecular activity. In the S_0_ state, cis-SA and cis-MS exhibit superior antioxidant activities. However, in the S_1_ state, trans-SA and cis-MS demonstrate enhanced radical-scavenging abilities in Eac and water, while cis-SA and cis-MS dominate in MeOH. This study bridges a critical gap in understanding the modulation of antioxidant properties through photoisomerization and solvent polarity. These structure–property relationships provide a molecular-level foundation for rational formulation design in applications requiring effective antioxidant protection. Our findings suggest that environments or processing conditions that favor the formation of cis-isomers (such as exposure to specific light wavelengths or selection of appropriate solvents) could potentially enhance the antioxidant efficacy of SA-based formulations. For instance, controlled photoisomerization could be integrated into processing protocols for functional foods or topical formulations to optimize antioxidant performance. Valuable practical insights are provided for applications in food science, cosmetic formulations, and drug development. This study focuses on the specific configurations and solvent environments of SA and its methyl ester. Future work could expand to a broader range of structural analogs and more diverse environmental conditions to establish a more comprehensive structure–activity relationship.

## Figures and Tables

**Table 1:** Dihedral angles (°) of studied compounds in S_0_ and S_1_ states.

			cis-SA	trans-SA	cis-MS	trans-MS
S_0_	Eac	D_3-1-2-4_	0.120	−179.944	1.053	−179.923
		D_1-2-4-o_	−0.195	−0.006	5.506	−0.022
	MeOH	D_3-1-2-4_	0.142	−179.990	2.851	−179.934
		D_1-2-4-o_	−0.241	0.291	18.798	0.060
	Water	D_3-1-2-4_	0.145	179.993	2.938	−179.947
		D_1-2-4-o_	−0.253	0.350	18.855	0.085
S_1_	Eac	D_3-1-2-4_	−0.013	−179.990	11.79	179.991
		D_1-2-4-o_	−0.047	0.019	2.291	−0.010
	MeOH	D_3-1-2-4_	0.525	−179.706	13.199	−179.693
		D_1-2-4-o_	0.163	−0.254	2.551	−0.096
	Water	D_3-1-2-4_	0.638	−179.689	13.445	−179.714
		D_1-2-4-o_	0.181	−0.101	2.603	−0.118

**Table 2:** The NPA charges of the hydrogen atom on the hydroxyl group of the investigated compounds.

			cis-SA	trans-SA	cis-MS	trans-MS
Eac	S_0_	H_1_	0.49140	0.49161	0.49071	0.49104
	S_1_		0.49708	0.50481	0.49373	0.50512
	S_0_	H_2_	0.48647	0.47932		
	S_1_		0.47101	0.46309		
MeOH	S_0_	H_1_	0.49371	0.49405	0.49261	0.49354
	S_1_		0.50263	0.50556	0.49666	0.50543
	S_0_	H_2_	0.48980	0.48564		
	S_1_		0.47428	0.47325		
Water	S_0_	H_1_	0.49405	0.49440	0.49283	0.49391
	S_1_		0.50607	0.50608	0.49713	0.50597
	S_0_	H_2_	0.49028	0.48658		
	S_1_		0.47759	0.47417		

**Table 3:** Several global descriptive parameters used to model antioxidant properties.

			IP (eV)	EA (eV)	η (eV)	ω (eV)	S (eV^−1^)	μ (eV)
cis-SA	Eac	S_0_	5.9671	1.5191	4.4480	1.5750	0.2248	−3.7431
		S_1_	5.8363	1.8024	4.0338	1.8081	0.2479	−3.8193
	MeOH	S_0_	5.7512	1.8169	3.9343	1.8197	0.2542	−3.7840
		S_1_	5.6010	2.1023	3.4987	2.1201	0.2858	−3.8517
	Water	S_0_	5.7225	1.8578	3.8646	1.8586	0.2588	−3.7902
		S_1_	5.5516	2.0998	3.4518	2.1201	0.2897	−3.8257
trans-SA	Eac	S_0_	6.0332	1.5276	4.5056	1.5859	0.2219	−3.7804
		S_1_	5.7569	1.7732	3.9837	1.7792	0.2510	−3.7651
	MeOH	S_0_	5.7841	1.8112	3.9729	1.8150	0.25107	−3.7976
		S_1_	5.6093	2.0858	3.5235	2.1007	0.2838	−3.8475
	Water	S_0_	5.7504	1.8498	3.9007	1.8511	0.2564	−3.8001
		S_1_	5.5752	2.1263	3.4489	2.1498	0.2900	−3.8508
cis-MS	Eac	S_0_	5.9706	1.5788	4.3918	1.6222	0.2277	−3.7747
		S_1_	5.1760	1.9203	3.2557	1.9334	0.3072	−3.5481
	MeOH	S_0_	5.7510	1.8325	3.9185	1.8346	0.2552	−3.7917
		S_1_	5.0366	2.2297	2.8069	2.3513	0.3563	−3.6331
	Water	S_0_	5.7225	1.8734	3.8491	1.8737	0.2598	−3.7980
		S_1_	5.0200	2.2730	2.7470	2.4203	0.3640	−3.6465
trans-MS	Eac	S_0_	5.9500	1.4242	4.5258	1.5019	0.2210	−3.6871
		S_1_	5.6675	1.6847	3.9828	1.6965	0.2511	−3.6761
	MeOH	S_0_	5.7271	1.7231	4.0039	1.7328	0.2498	−3.7251
		S_1_	5.5433	2.0227	3.5206	2.0325	0.2840	−3.7830
	Water	S_0_	5.6977	1.7643	3.9333	1.7695	0.2542	−3.7310
		S_1_	5.5127	2.0656	3.4471	2.0825	0.2901	−3.7891

## Data Availability

The original contributions presented in this study are included in the article/Supplementary Materials. Further inquiries can be directed to the corresponding authors.

## Supplementary Materials

The following supporting information can be downloaded at: https://www.mdpi.com/article/10.3390/antiox14060633/s1, Figure S1. Orbitals used in the active space for CASSCF(2,3) calculations of SA; Figure S2. Orbitals used in the active space for CASSCF(4,4) calculations of MS; Figure S3. Potential energy surface of SA scanned at the CAM-B3LYP/ 6-311+G(d,p) level; Figure S4. Potential energy surface of MS scanned at the CAM-B3LYP/ 6-311+G(d,p) level; Figure S5. The electrostatic potential distribution of the compounds in MeOH at S0 and S1 states; Figure S6. The electrostatic potential distribution of the compounds in Water at S0 and S1 states; Table S1. Optimized Cartesian coordinates of the S_0_ and S_1_ states of the cis-SA and trans-SA in Eac; Table S2. Optimized Cartesian coordinates of the S_0_ and S_1_ states of the cis-SA and trans-SA in MeOH; Table S3. Optimized Cartesian coordinates of the S_0_ and S1 states of the cis-SA and trans-SA in water; Table S4. Optimized Cartesian coordinates of the S0 and S1 states of the cis-MS and trans-MS in Eac; Table S5. Optimized Cartesian coordinates of the S0 and S1 states of the cis-MS and trans-MS in MeOH; Table S6. Optimized Cartesian coordinates of the S0 and S1 states of the cis-MS and trans-MS in water; Table S7. Calculated transition properties of cis-SA, trans-SA, cis-MS and trans-MS in three solvents; Table S8. Cartesian coordinates of conical intersections of SA in Eac; Table S9. Cartesian coordinates of conical intersections of SA in MeOH; Table S10. Cartesian coordinates of conical intersections of SA in water; Table S11. Cartesian coordinates of conical intersections of MS in Eac; Table S12. Cartesian coordinates of conical intersections of MS in MeOH; Table S13. Cartesian coordinates of conical intersections of MS in water.

## References

[1] Barayeu U., Schilling D., Eid M., da Silva T.N.X., Schlicker L., Mitreska N., Zapp C., Gräter F., Miller A.K., Kappl R. (2023). Hydropersulfides inhibit lipid peroxidation and ferroptosis by scavenging radicals. Nat. Chem. Biol..

[2] Treml J., Smejkal K. (2016). Flavonoids as Potent Scavengers of Hydroxyl Radicals. Compr. Rev. Food Sci. Food Saf..

[3] Cao B.F., Li K., Chen C.G., Shi Y. (2025). Effects of functional groups on ESIPT and antioxidant activity of apigenin: A non-existent enol* state fluorescein. Spectrochim. Acta Part A.

[4] Sies H. (2015). Oxidative stress: A concept in redox biology and medicine. Redox Biol..

[5] Niggeweg R., Michael A.J., Martin C. (2004). Engineering plants with increased levels of the antioxidant chlorogenic acid. Nat. Biotechnol..

[6] Rico D., Martín-Diana A.B., Barat J.M., Barry-Ryan C. (2007). Extending and measuring the quality of fresh-cut fruit and vegetables: A review. Trends Food Sci. Technol..

[7] Nićiforović N., Abramovič H. (2014). Sinapic Acid and Its Derivatives: Natural Sources and Bioactivity. Compr. Rev. Food Sci. Food Saf..

[8] Zhao X., Luo J., Yang S., Han K. (2019). New Insight into the Photoprotection Mechanism of Plant Sunscreens: Adiabatic Relaxation Competing with Nonadiabatic Relaxation in the cis → trans Photoisomerization of Methyl Sinapate. J. Phys. Chem. Lett..

[9] Mathew S., Abraham T.E., Zakaria Z.A. (2015). Reactivity of phenolic compounds towards free radicals under in vitro conditions. J. Food Sci. Technol..

[10] Yoshinaga M., Toldo J.M., Rocha W.R., Barbatti M. (2024). Photoisomerization pathways of *trans*-resveratrol. Phys. Chem. Chem. Phys..

[11] Welc-Stanowska R., Pietras R., Mielecki B., Sarewicz M., Luchowski R., Widomska J., Grudzinski W., Osyczka A., Gruszecki W.I. (2023). How Do Xanthophylls Protect Lipid Membranes from Oxidative Damage?. J. Phys. Chem. Lett..

[12] Wang L., Wang L., Zhang Y., Sun C., Huang Z. (2025). Influence of cis–trans isomerization induced by photoexcitation on the antioxidant properties of piceatannol and its derivatives. Chem. Phys. Lett..

[13] Gangadasu B., Reddy M.J.R., Ravinder M., Kumar S.B., Raju B.C., Kumar K.P., Murthy U.S.N., Rao V.J. (2009). Synthesis, photochemical E (trans)→Z (cis) isomerization and antimicrobial activity of 2-chloro-5-methylpyridine-3-olefin derivatives. Eur. J. Med. Chem..

[14] Liu X., Osawa T. (2007). Cis astaxanthin and especially 9-cis astaxanthin exhibits a higher antioxidant activity in vitro compared to the all-trans isomer. Biochem. Biophys. Res. Commun..

[15] Zheng X., Huang Q. (2022). Assessment of the antioxidant activities of representative optical and geometric isomers of astaxanthin against singlet oxygen in solution by a spectroscopic approach. Food Chem..

[16] Bermúdez-Oria A., Castejón M.L., Rubio-Senent F., Fernández-Prior Á., Rodríguez-Gutiérrez G., Fernández-Bolaños J. (2024). Isolation and structural determination of cis- and trans-p-coumaroyl-secologanoside (comselogoside) from olive oil waste (alperujo). Photoisomerization with ultraviolet irradiation and antioxidant activities. Food Chem..

[17] Joshi N.K., Fuyuki M., Wada A. (2014). Polarity Controlled Reaction Path and Kinetics of Thermal Cis-to-Trans Isomerization of 4-Aminoazobenzene. J. Phys. Chem. B.

[18] Kohn W., Sham L.J. (1965). Quantum density oscillations in an inhomogeneous electron gas. Phys. Rev..

[19] Lee C., Yang W., Parr R.G. (1988). Development of the Colle-Salvetti correlation-energy formula into a functional of the electron density. Phys. Rev. B.

[20] Stratmann R.E., Scuseria G.E., Frisch M.J. (1998). An efficient implementation of time-dependent density-functional theory for the calculation of excitation energies of large molecules. J. Chem. Phys..

[21] Matsuzawa N.N., Ishitani A., Dixon D.A., Uda T. (2001). Time-dependent density functional theory calculations of photoabsorption spectra in the vacuum ultraviolet region. J. Phys. Chem. A.

[22] Li Q., Ding Q., Lin W., Wang J., Chen M., Sun M. (2017). Surface-enhanced Raman scattering of pyrazine on Au5Al5 bimetallic nanoclusters. RSC Adv..

[23] Becke A.D. (1988). Density-functional exchange-energy approximation with correct asymptotic behavior. Phys. Rev. A.

[24] Merchán M., Roos B.O. (1991). The electron affinity of NH2: A restricted active-space SCF multi-reference CI study. Chem. Phys. Lett..

[25] Malmqvist P.Å., Roos B.O., Schimmelpfennig B. (2002). The restricted active space (RAS) state interaction approach with spin–orbit coupling. Chem. Phys. Lett..

[26] Fdez. Galván I., Vacher M., Alavi A., Angeli C., Aquilante F., Autschbach J., Bao J.J., Bokarev S.I., Bogdanov N.A., Carlson R.K. (2019). OpenMolcas: From Source Code to Insight. J. Chem. Theory Comput..

[27] Li Manni G., Fdez. Galván I., Alavi A., Aleotti F., Aquilante F., Autschbach J., Avagliano D., Baiardi A., Bao J.J., Battaglia S. (2023). The OpenMolcas Web: A Community-Driven Approach to Advancing Computational Chemistry. J. Chem. Theory Comput..

[28] Roos B.O., Taylor P.R., Sigbahn P.E.M. (1980). A complete active space SCF method (CASSCF) using a density matrix formulated super-CI approach. Chem. Phys..

[29] Bearpark M.J., Ogliaro F., Vreven T., Boggio-Pasqua M., Frisch M.J., Larkin S.M., Morrison M., Robb M.A. (2007). CASSCF calculations for photoinduced processes in large molecules: Choosing when to use the RASSCF, ONIOM and MMVB approximations. J. Photochem. Photobiol. A.

[30] Tomasello G., Garavelli M., Orlandi G. (2013). Tracking the stilbene photoisomerization in the S_1_ state using RASSCF. Phys. Chem. Chem. Phys..

[31] Boggio-Pasqua M., Robb M.A., Bearpark M.J. (2005). Photostability via a sloped conical intersection: A CASSCF and RASSCF study of pyracylene. J. Phys. Chem. A.

[32] Krausbeck F., Mendive-Tapia D., Thom A.J.W., Bearpark M.J. (2014). Choosing RASSCF orbital active spaces for multiple electronic states. Comput. Theor. Chem..

[33] Roos B.O., Andersson K. (1995). Multiconfigurational perturbation theory with level shift—the Cr2 potential revisited. Chem. Phys. Lett..

[34] Roos B.O., Andersson K., Fülscher M.P., Serrano-Andrés L., Pierloot K., Merchán M., Molina V. (1996). Applications of level shift corrected perturbation theory in electronic spectroscopy. J. Mol. Struct. THEOCHEM.

[35] Park J.W. (2019). Single-State Single-Reference and Multistate Multireference Zeroth-Order Hamiltonians in MS-CASPT2 and Conical Intersections. J. Chem. Theory Comput..

[36] Wang W., Li Z.-W., Wang X.-T.-H., Xia S.-H. (2024). Photocyclization and Photoisomerization Mechanisms of an Indolylfulgide Derivative in Acetonitrile Solution by Using the QM (MS-CASPT2)/MM Method. J. Phys. Chem. A.

[37] Lu T., Chen F. (2012). Multiwfn: A multifunctional wavefunction analyzer. J. Comput. Chem..

[38] Lu T. (2024). A comprehensive electron wavefunction analysis toolbox for chemists, Multiwfn. J. Chem. Phys..

[39] Roohi H., Mohtamedifar N., Hejazi F. (2014). Intramolecular photoinduced proton transfer in 2-(2′-hydroxyphenyl)benzazole family: A TD-DFT quantum chemical study. Chem. Phys..

[40] Rajan V.K., Muraleedharan K. (2017). A computational investigation on the structure, global parameters and antioxidant capacity of a polyphenol, Gallic acid. Food Chem..

[41] Wang L.L., Yang F.J., Zhao X.H., Li Y.Z. (2019). Effects of nitro- and amino-group on the antioxidant activity of genistein: A theoretical study. Food Chem..

[42] Jeevitha D., Sadasivam K., Praveena R., Jayaprakasam R. (2016). DFT study of glycosyl group reactivity in quercetin derivatives. J. Mol. Struct..

[43] Sadasivam K., Kumaresan R. (2011). Antioxidant behavior of mearnsetin and myricetin flavonoid compounds—A DFT study. Spectrochim. Acta Part A.

[44] Martínez-Araya J.I. (2024). The dual descriptor potential. J. Math. Chem..

[45] Halliday G.M., Byrne S.N., Damian D.L. (2011). Ultraviolet A Radiation: Its Role in Immunosuppression and Carcinogenesis. Semin. Cutaneous Med. Surg..

[46] Frisch M.J., Trucks G.W., Schlegel H.B., Scuseria G.E., Robb M.A., Cheeseman J.R., Scalmani G., Barone V., Mennucci B., Petersson G.A. (2016). Gaussian 16, Revision B.03.

[47] Battie C., Jitsukawa S., Bernerd F., Del Bino S., Marionnet C., Verschoore M. (2014). New insights in photoaging, UVA induced damage and skin types. Exp. Dermatol..

[48] Brem R., Guven M., Karran P. (2017). Oxidatively-generated damage to DNA and proteins mediated by photosensitized UVA. Free Radical Biol. Med..

[49] Liu X.D., Feng J.K., Ren A.M., Yang L., Yang B., Ma Y.G. (2006). Theoretical studies of electronic structures, absorption and emission spectra in cyclometalated phenylpyridine Ir(III) complex and its derivatives using density functional theory. Opt. Mater..

[50] Chen C., Wang K.Y., Jiang P., Song G.L., Zhu H.J. (2012). Synthesis, crystal structures and photophysical properties of novel copper(I) complexes with 4-diphenylphosphino-1,5-naphthyridine ligands. Inorg. Chem. Commun..

[51] Narsaria A.K., Poater J., Guerra C.F., Ehlers A.W., Hamlin T.A., Lammertsma K., Bickelhaupt F.M. (2020). Distortion-Controlled Redshift of Organic Dye Molecules. Chem.-Eur. J..

[52] Yang Y., Li D., Li C., Liu Y., Jiang K. (2019). Asymmetric substitution changes the UV-induced nonradiative decay pathway and the spectra behaviors of β-diketones. Spectrochim. Acta Part A.

[53] Li Y.Y., Wang L., Guo X.G., Zhang J.L. (2015). A CASSCF/CASPT2 Insight into Excited-State Intramolecular Proton Transfer of Four Imidazole Derivatives. J. Comput. Chem..

[54] Shi Y.N., Zhao X.Y., Wang C., Wang Y., Zhang S., Li P., Feng X., Jin B., Yuan M.H., Cui S. (2020). Ultrafast Nonadiabatic Photoisomerization Dynamics Mechanism for the UV Photoprotection of Stilbenoids in Grape Skin. Chem.-Asian J..

[55] Zhou P.W., Liu J.Y., Han K.L., He G.Z. (2014). The Photoisomerization of 11-cis-Retinal Protonated Schiff Base in Gas Phase: Insight from Spin-Flip Density Functional Theory. J. Comput. Chem..

[56] Galego J., Garcia-Vidal F.J., Feist J. (2016). Suppressing photochemical reactions with quantized light fields. Nat. Commun..

[57] Polli D., Altoè P., Weingart O., Spillane K.M., Manzoni C., Brida D., Tomasello G., Orlandi G., Kukura P., Mathies R.A. (2010). Conical intersection dynamics of the primary photoisomerization event in vision. Nature.

[58] von Conta A., Tehlar A., Schletter A., Arasaki Y., Takatsuka K., Wörner H.J. (2018). Conical-intersection dynamics and ground-state chemistry probed by extreme-ultraviolet time-resolved photoelectron spectroscopy. Nat. Commun..

[59] Oliver T.A.A., Fleming G.R. (2015). Following Coupled Electronic-Nuclear Motion through Conical Intersections in the Ultrafast Relaxation of β-Apo-8′-carotenal. J. Phys. Chem. B.

[60] Andersson K., Malmqvist P.A., Roos B.O., Sadlej A.J., Wolinski K. (1990). Second-order perturbation theory with a CASSCF reference function. J. Phys. Chem..

[61] Olsen J., Roos B.O., Jo/rgensen P., Jensen H.J.r.A. (1988). Determinant based configuration interaction algorithms for complete and restricted configuration interaction spaces. J. Chem. Phys..

[62] Medimagh M., Ben Mleh C., Issaoui N., Kazachenko A.S., Roisnel T., Al-Dossary O.M., Marouani H., Bousiakoug L.G. (2023). DFT and molecular docking study of the effect of a green solvent (water and DMSO) on the structure, MEP, and FMOs of the 1-ethylpiperazine-1,4-diium bis(hydrogenoxalate) compound. J. Mol. Liq..

[63] Uludag N., Serdaroglu G., Sugumar P., Rajkumar P., Colak N., Ercag E. (2022). Synthesis of thiophene derivatives: Substituent effect, antioxidant activity, cyclic voltammetry, molecular docking, DFT, and TD-DFT calculations. J. Mol. Struct..

[64] Zhang Y.J., Shang C.J., Sun C.F., Wang L.L. (2024). Simultaneously regulating absorption capacities and antioxidant activities of four stilbene derivatives utilizing substitution effect: A theoretical and experimental study against UVB radiation. Spectrochim. Acta Part A.

[65] Madala N.E., Kabanda M.M. (2021). LC-MS based validation and DFT investigation on the antioxidant properties of clovamide: ^•^OH and ^•^OOH scavenging and Cu(II) chelation mechanisms. J. Mol. Struct..

[66] Cao B., Li Y., Zhou Q., Li B., Su X., Yin H., Shi Y. (2021). Synergistically improving myricetin ESIPT and antioxidant activity via dexterously trimming atomic electronegativity. J. Mol. Liq..

[67] Hu G., Guo M., Li Q., Zhao J., Zhang L., Yang J., Yin H., Han J., Shi Y. (2025). The substituent effect on the ESIPT and antioxidant activity of dual proton-transfer-site salicylaldehyde azine derivatives. Chem. Phys. Lett..

