# Supplementary material for: The Effect of Photoisomerization on the Antioxidant Properties of Sinapic Acid and Methyl Sinapate in Different Solvents: A DFT/TD-DFT Study
Source: Antioxidants (Basel). 2025 May 25;14(6):633. doi: 10.3390/antiox14060633 (PMC12189039; doi:10.3390/antiox14060633)
Supplement: Supplementary file 1 [file antioxidants-14-00633-s001.zip › antioxidants-3610457-supplementary.pdf]

Supplementary Materials

The Effect of Photoisomerization on the Antioxidant Properties of Sinapic Acid and Methyl Sinapate in Different Solvents: A DFT/TD-DFT Study

Lei Wang [1], Chaofan Sun [1,*] and Lingling Wang [2,3,4,*]

[1] College of Science, Northeast Forestry University, Harbin 150040, China; wangl@nefu.edu.cn
[2] College of Chemistry, Chemical Engineering and Resource Utilization, Northeast Forestry University, Harbin 150040, China
[3] Key Laboratory of Forest Plant Ecology, Ministry of Education, Northeast Forestry University, Harbin 150040, China
[4] Heilongjiang Provincial Key Laboratory of Ecological Utilization of Forestry-Based Active Substances, Northeast Forestry University, Harbin 150040, China
* Correspondence: cfsun@nefu.edu.cn (C.S.); llwang@nefu.edu.cn (L.W.)

Table S1. Optimized Cartesian coordinates of the S_0 and S_1 states of the cis-SA and trans-SA in Eac.

S_0	cis-SA	C	-0.84046	-1.60453	-0.21346
		C	-2.04854	-0.91746	-0.23592
		C	-2.04783	0.479225	-0.16432
		C	-0.81757	1.161143	-0.0587
		C	0.384664	0.47696	-0.04216
		C	0.391952	-0.93181	-0.12542
		O	-3.21847	-1.62167	-0.38096
		C	-4.10009	-1.5886	0.755443
		O	-0.96321	2.519008	0.007676
		C	0.216475	3.324376	0.08592
		O	-3.21503	1.166973	-0.20363
		C	1.585226	-1.76655	-0.12224
		C	2.922731	-1.55839	-0.04243
		C	3.700778	-0.3218	0.080693
		O	3.31625	0.832123	0.137992
		O	5.027816	-0.61407	0.130384
		H	-0.88053	-2.68542	-0.27766
		H	1.322549	1.002243	0.030946
		H	-4.94401	-2.22882	0.501535
		H	-4.4548	-0.57474	0.952346
		H	-3.59578	-1.98412	1.643518
		H	-0.12945	4.355149	0.122353
		H	0.849156	3.17664	-0.79415
		H	0.78803	3.093457	0.989585
		H	-2.99746	2.108884	-0.14077
		H	1.333522	-2.82177	-0.20445
		H	3.546284	-2.44392	-0.07082
		H	5.499644	0.228064	0.211456
	trans-SA	C	-0.44545	-1.40331	-0.2066
		C	-1.81689	-1.16408	-0.24037
		C	-2.28873	0.148701	-0.16084
		C	-1.36504	1.211111	-0.03499
		C	-0.00329	0.968814	-0.0068
		C	0.477122	-0.35487	-0.09817

		O	-2.67555	-2.22072	-0.40525
		C	-3.53905	-2.49576	0.712913
		O	-1.96902	2.433632	0.035223
		C	-1.14905	3.600566	0.130252
		O	-3.61945	0.400847	-0.21332
		C	1.893534	-0.68162	-0.07651
		C	2.941851	0.154944	0.039242
		C	4.31638	-0.38246	0.03862
		O	4.611289	-1.55242	-0.05976
		O	5.313524	0.536056	0.160695
		H	-0.1102	-2.43105	-0.27706
		H	0.688824	1.793604	0.080433
		H	-4.11057	-3.382	0.440655
		H	-4.21951	-1.66312	0.902084
		H	-2.94774	-2.70391	1.610736
		H	-1.83508	4.443958	0.164409
		H	-0.49724	3.691824	-0.74388
		H	-0.54562	3.580979	1.042849
		H	-3.73756	1.359447	-0.14039
		H	2.134741	-1.73808	-0.16455
		H	2.811546	1.227873	0.135709
		H	4.95367	1.42903	0.227909
S_1	cis-SA	C	-0.92375	-1.59851	-0.20753
		C	-2.10694	-0.88075	-0.23121
		C	-2.06607	0.519929	-0.16808
		C	-0.77901	1.142729	-0.06862
		C	0.420036	0.403639	-0.05502
		C	0.381318	-0.98449	-0.13456
		O	-3.29408	-1.54762	-0.36803
		C	-4.17669	-1.48214	0.769534
		O	-0.84129	2.473441	-0.00537
		C	0.369525	3.25785	0.086412
		O	-3.1991	1.245743	-0.21121
		C	1.557491	-1.84363	-0.14109
		C	2.922635	-1.56947	-0.04828

	C	3.670668	-0.35594	0.086297
	O	3.291906	0.828653	0.159022
	O	5.033639	-0.61222	0.137421
	H	-1.00551	-2.67838	-0.25894
	H	1.378229	0.900799	0.016814
	H	-5.03336	-2.10569	0.519259
	H	-4.5089	-0.45856	0.954671
	H	-3.68048	-1.88054	1.660088
	H	0.037287	4.290826	0.124072
	H	0.992748	3.083207	-0.79165
	H	0.915955	2.993106	0.992582
	H	-2.97397	2.186488	-0.15103
	H	1.321942	-2.89894	-0.23111
	H	3.560774	-2.44636	-0.08269
	H	5.451416	0.25467	0.226072
trans-SA	C	-0.47723	-1.49327	-0.00027
	C	-1.86139	-1.16425	0.000059
	C	-2.28092	0.19912	-4.1E-05
	C	-1.28227	1.191959	-0.00049
	C	0.059171	0.86292	-0.00078
	C	0.505814	-0.51726	-0.0006
	O	-2.66943	-2.2177	0.000474
	C	-4.1136	-2.13787	0.000921
	O	-1.80229	2.450664	-0.00065
	C	-0.90851	3.570875	-0.00025
	O	-3.58713	0.542287	0.000309
	C	1.90392	-0.87083	-0.0008
	C	2.963837	0.031701	0.000691
	C	4.328531	-0.41721	0.000149
	O	4.710045	-1.58669	-0.00192
	O	5.309151	0.567836	0.002107
	H	-0.22783	-2.54612	-0.00026
	H	0.795201	1.652655	-0.00149
	H	-4.44355	-3.1725	0.001314
	H	-4.4594	-1.61669	-0.89074

H	-4.45882	-1.61612	0.892457
H	-1.54351	4.45347	0.000143
H	-0.28175	3.566443	-0.89625
H	-0.28163	3.565691	0.895675
H	-3.63026	1.513002	0.00072
H	2.146149	-1.9266	-0.00201
H	2.783314	1.101858	0.002646
H	4.895217	1.438434	0.003658

Table S2. Optimized Cartesian coordinates of the S_0 and S_1 states of the cis-SA and trans-SA in MeOH.

S_0	cis-SA	C	-0.84111	-1.602839	-0.218523
		C	-2.048674	-0.915091	-0.240872
		C	-2.049343	0.481361	-0.167578
		C	-0.819542	1.164678	-0.06188
		C	0.382804	0.479737	-0.045725
		C	0.391039	-0.929285	-0.129505
		O	-3.221812	-1.615826	-0.384974
		C	-4.082672	-1.606969	0.76928
		O	-0.965889	2.521193	0.005523
		C	0.212309	3.328931	0.093221
		O	-3.218254	1.165714	-0.205352
		C	1.584223	-1.764745	-0.126091
		C	2.921927	-1.557818	-0.044587
		C	3.701676	-0.322426	0.081383
		O	3.316766	0.831828	0.139381
		O	5.027321	-0.616081	0.133411
		H	-0.880095	-2.683792	-0.282149
		H	1.320331	1.005585	0.027821
		H	-4.93894	-2.229611	0.51329
		H	-4.421066	-0.594965	1.001743
		H	-3.566144	-2.033589	1.635278
		H	-0.135997	4.358596	0.132552
		H	0.848892	3.186646	-0.784851
		H	0.779109	3.095123	0.999041

		H	-3.006084	2.108624	-0.135766
		H	1.332145	-2.819645	-0.209527
		H	3.543696	-2.44461	-0.073141
		H	5.503274	0.223798	0.217042
	trans-SA	C	-0.44909	-1.40494	-0.21218
		C	-1.81967	-1.16203	-0.24534
		C	-2.28976	0.151119	-0.16451
		C	-1.36479	1.212988	-0.03926
		C	-0.00336	0.967546	-0.01179
		C	0.474817	-0.35719	-0.10341
		O	-2.68522	-2.21515	-0.40815
		C	-3.52053	-2.50309	0.729427
		O	-1.96595	2.434992	0.031915
		C	-1.14366	3.601524	0.136986
		O	-3.62038	0.402947	-0.21474
		C	1.890808	-0.68533	-0.08161
		C	2.938142	0.15246	0.038727
		C	4.312666	-0.38032	0.039018
		O	4.613052	-1.55039	-0.06772
		O	5.307305	0.536796	0.171504
		H	-0.11503	-2.43319	-0.28157
		H	0.689707	1.791477	0.075427
		H	-4.10882	-3.3786	0.45817
		H	-4.18671	-1.66698	0.951861
		H	-2.90653	-2.7323	1.606324
		H	-1.82934	4.44481	0.174979
		H	-0.48982	3.697058	-0.7348
		H	-0.5428	3.573985	1.05066
		H	-3.74042	1.361117	-0.13503
		H	2.130184	-1.74195	-0.17301
		H	2.806886	1.224363	0.139531
		H	4.9488	1.430164	0.24853
S_1	cis-SA	C	-0.9221	-1.60205	-0.2075
		C	-2.10204	-0.88989	-0.23362
		C	-2.06239	0.516804	-0.17011

	C	-0.77924	1.150778	-0.06626
	C	0.41572	0.419824	-0.04869
	C	0.380281	-0.97471	-0.12859
	O	-3.293	-1.54853	-0.3775
	C	-4.16288	-1.50942	0.772875
	O	-0.86086	2.482041	-0.00323
	C	0.339621	3.277717	0.095003
	O	-3.1957	1.232541	-0.22043
	C	1.551064	-1.83118	-0.1305
	C	2.921463	-1.56443	-0.04582
	C	3.680261	-0.35608	0.083285
	O	3.311296	0.830318	0.160724
	O	5.041032	-0.62549	0.121989
	H	-0.99377	-2.68231	-0.26019
	H	1.371948	0.918226	0.026226
	H	-5.03284	-2.10915	0.510823
	H	-4.47518	-0.48749	0.997529
	H	-3.66268	-1.94695	1.642194
	H	-0.00149	4.307883	0.129969
	H	0.970182	3.109961	-0.77967
	H	0.88318	3.020495	1.005541
	H	-2.97865	2.176357	-0.15743
	H	1.314569	-2.88684	-0.21465
	H	3.552139	-2.44681	-0.08292
	H	5.471913	0.235481	0.209601
trans-SA	C	-0.52164	-1.44813	-0.19139
	C	-1.86476	-1.16243	-0.22455
	C	-2.28892	0.194645	-0.1567
	C	-1.30449	1.225476	-0.02381
	C	0.049493	0.931184	0.013998
	C	0.481563	-0.41983	-0.07958
	O	-2.76949	-2.16577	-0.40104
	C	-3.69721	-2.39069	0.683756
	O	-1.85643	2.451204	0.03982
	C	-1.00995	3.609058	0.145904

		O	-3.58988	0.493712	-0.24999
		C	1.864205	-0.79895	-0.05056
		C	2.946013	0.080366	0.017242
		C	4.301054	-0.40744	0.034892
		O	4.63852	-1.59198	-0.00127
		O	5.308162	0.537207	0.098772
		H	-0.21976	-2.48611	-0.25685
		H	0.768904	1.727684	0.121249
		H	-4.29873	-3.24695	0.384901
		H	-4.34072	-1.52423	0.84209
		H	-3.15059	-2.62629	1.601367
		H	-1.68389	4.460813	0.16922
		H	-0.34834	3.674266	-0.72072
		H	-0.4246	3.56627	1.067043
		H	-3.69392	1.458513	-0.1919
		H	2.091873	-1.8576	-0.09481
		H	2.78831	1.153528	0.048251
		H	4.932177	1.425458	0.124628

Table S3. Optimized Cartesian coordinates of the S_0 and S_1 states of the cis-SA and trans-SA in water.

S_0	cis-SA	C	-0.84122	-1.6026	-0.21926
		C	-2.04869	-0.91473	-0.2416
		C	-2.04956	0.481682	-0.16809
		C	-0.81982	1.165203	-0.06243
		C	0.382528	0.480135	-0.04632
		C	0.390895	-0.92893	-0.13013
		O	-3.22231	-1.61495	-0.3855
		C	-4.08007	-1.60964	0.771326
		O	-0.96624	2.521525	0.005062
		C	0.211731	3.329561	0.094505
		O	-3.21872	1.165527	-0.20565
		C	1.584063	-1.7645	-0.12664
		C	2.921794	-1.55774	-0.04488
		C	3.701779	-0.32252	0.081475

	O	3.316812	0.831787	0.139453
	O	5.027214	-0.61636	0.133895
	H	-0.88007	-2.68356	-0.28272
	H	1.32001	1.006054	0.027275
	H	-4.93814	-2.22972	0.515186
	H	-4.41598	-0.598	1.00893
	H	-3.56176	-2.04072	1.633983
	H	-0.1369	4.359066	0.134312
	H	0.849073	3.188171	-0.78315
	H	0.777626	3.095197	1.000732
	H	-3.00736	2.108561	-0.13483
	H	1.331945	-2.81935	-0.21021
	H	3.543298	-2.44472	-0.07339
	H	5.503732	0.22321	0.21787
trans-SA	C	-0.4498	-1.40525	-0.21315
	C	-1.82022	-1.16164	-0.24619
	C	-2.28994	0.151594	-0.16512
	C	-1.36469	1.213295	-0.03999
	C	-0.00333	0.967241	-0.01265
	C	0.474402	-0.35768	-0.10434
	O	-2.68695	-2.21413	-0.40862
	C	-3.51753	-2.50422	0.732091
	O	-1.96528	2.435296	0.031358
	C	-1.14251	3.601648	0.138242
	O	-3.62055	0.40345	-0.21489
	C	1.890325	-0.68613	-0.08246
	C	2.937477	0.151906	0.038309
	C	4.312056	-0.38003	0.039078
	O	4.61342	-1.55009	-0.06865
	O	5.306161	0.537019	0.173203
	H	-0.11602	-2.43361	-0.28234
	H	0.689952	1.790971	0.074676
	H	-4.10885	-3.37774	0.460965
	H	-4.18099	-1.66746	0.960225
	H	-2.89983	-2.73723	1.605328

		H	-1.82805	4.444981	0.177004
		H	-0.48833	3.697975	-0.73315
		H	-0.54204	3.572623	1.052068
		H	-3.74081	1.361532	-0.13391
		H	2.129392	-1.74281	-0.17406
		H	2.805987	1.223673	0.139343
		H	4.947675	1.430369	0.251686
S_1	cis-SA	C	-0.92166	-1.60257	-0.20771
		C	-2.10143	-0.89113	-0.23371
		C	-2.06213	0.516244	-0.17047
		C	-0.77945	1.151725	-0.06666
		C	0.415147	0.421767	-0.04922
		C	0.380349	-0.97365	-0.12901
		O	-3.29268	-1.54912	-0.37747
		C	-4.16089	-1.51229	0.774602
		O	-0.86334	2.482884	-0.00367
		C	0.335784	3.280087	0.095007
		O	-3.19554	1.230626	-0.22135
		C	1.550527	-1.82955	-0.13129
		C	2.921654	-1.56356	-0.04704
		C	3.681355	-0.35601	0.083914
		O	3.313231	0.83041	0.164278
		O	5.041969	-0.62657	0.120767
		H	-0.99228	-2.68289	-0.26003
		H	1.371088	0.92047	0.025855
		H	-5.03263	-2.109	0.511668
		H	-4.47034	-0.49046	1.003463
		H	-3.66015	-1.95417	1.641342
		H	-0.00657	4.309819	0.130355
		H	0.966658	3.113744	-0.77977
		H	0.879569	3.023449	1.005623
		H	-2.97974	2.174861	-0.15778
		H	1.314033	-2.88516	-0.21605
		H	3.551559	-2.44644	-0.08591
		H	5.474144	0.233597	0.210309

	trans-SA	C	-0.52175	-1.44746	-0.1935
		C	-1.86484	-1.16249	-0.22551
		C	-2.28983	0.194095	-0.15748
		C	-1.30557	1.225815	-0.02658
		C	0.04877	0.932304	0.008962
		C	0.481537	-0.41822	-0.08404
		O	-2.77013	-2.16627	-0.39927
		C	-3.68961	-2.3943	0.692001
		O	-1.85795	2.450708	0.037638
		C	-1.01259	3.609749	0.14326
		O	-3.59109	0.491694	-0.24713
		C	1.863927	-0.79702	-0.05727
		C	2.946098	0.081393	0.021828
		C	4.300728	-0.40641	0.035584
		O	4.638896	-1.59072	-0.01628
		O	5.308012	0.536769	0.114452
		H	-0.21929	-2.48537	-0.25776
		H	0.767664	1.729856	0.111715
		H	-4.29415	-3.24915	0.395136
		H	-4.33125	-1.52784	0.858105
		H	-3.13619	-2.63308	1.604683
		H	-1.68782	4.460325	0.169974
		H	-0.35378	3.677177	-0.72528
		H	-0.4245	3.565998	1.062541
		H	-3.69664	1.456332	-0.18796
		H	2.091479	-1.85532	-0.11048
		H	2.788602	1.153988	0.067646
		H	4.932621	1.424908	0.152875

Table S4. Optimized Cartesian coordinates of the S_0 and S_1 states of the cis-MS and trans-MS in Eac.

S_0	cis-MS	C	1.036595	-1.59371	0.171248
		C	2.314374	-1.05018	0.230967
		C	2.470486	0.338707	0.182114
		C	1.328006	1.156741	0.056489

	C	0.056207	0.615233	0.00133
	C	-0.11149	-0.78575	0.066485
	O	3.392501	-1.88415	0.398387
	C	4.313659	-1.94046	-0.70403
	O	1.633448	2.488945	0.011875
	C	0.561785	3.430937	-0.08712
	O	3.702636	0.895501	0.260767
	C	-1.38351	-1.49409	0.032714
	C	-2.70177	-1.16943	-0.03332
	C	-3.35922	0.140661	-0.12044
	O	-2.81375	1.220942	-0.24421
	O	-4.72028	0.163518	-0.07158
	C	-5.52497	-1.01081	0.120703
	H	0.948229	-2.67239	0.222915
	H	-0.81595	1.24192	-0.09348
	H	5.069537	-2.67405	-0.427
	H	4.787604	-0.97203	-0.8741
	H	3.802861	-2.26959	-1.61461
	H	1.030281	4.412598	-0.0996
	H	-0.10795	3.351378	0.773305
	H	-0.00569	3.280417	-1.00935
	H	3.585685	1.856267	0.207376
	H	-1.22961	-2.56992	0.084704
	H	-3.36579	-2.02247	-0.02109
	H	-6.54975	-0.64838	0.172785
	H	-5.275	-1.51837	1.054562
	H	-5.43189	-1.70052	-0.72118
trans-MS	C	-0.90351	-1.41165	-0.21055
	C	-2.2716	-1.15063	-0.237
	C	-2.72303	0.168847	-0.15579
	C	-1.78223	1.216013	-0.03509
	C	-0.42406	0.952445	-0.01443
	C	0.035721	-0.37804	-0.10813
	O	-3.14798	-2.19456	-0.39572
	C	-4.00775	-2.45438	0.728511

		O	-2.36566	2.448885	0.039033
		C	-1.52481	3.601049	0.132271
		O	-4.05112	0.442104	-0.20105
		C	1.449978	-0.72249	-0.09477
		C	2.502741	0.108387	0.008468
		C	3.872831	-0.42502	0.002551
		O	4.191849	-1.59414	-0.08923
		O	4.776728	0.576563	0.11572
		C	6.160105	0.175651	0.121284
		H	-0.58497	-2.4447	-0.28224
		H	0.2819	1.765755	0.069703
		H	-4.59653	-3.33086	0.461153
		H	-4.67251	-1.61016	0.922371
		H	-3.41379	-2.67219	1.62238
		H	-2.19568	4.456388	0.171506
		H	-0.87547	3.682645	-0.74462
		H	-0.91699	3.568937	1.041573
		H	-4.15253	1.402434	-0.12724
		H	1.677792	-1.78232	-0.17859
		H	2.404024	1.182807	0.098504
		H	6.731351	1.09628	0.215428
		H	6.413456	-0.33869	-0.80744
		H	6.36302	-0.48825	0.963531
S_1	cis-MS	C	-1.09151	-1.70873	-0.327458
		C	-2.2474	-0.92834	-0.025861
		C	-2.16029	0.479245	0.021478
		C	-0.94189	1.072517	-0.325603
		C	0.171953	0.304489	-0.690086
		C	0.124787	-1.13289	-0.606689
		O	-3.33257	-1.66636	0.237404
		C	-4.61192	-1.09831	0.590027
		O	-0.96659	2.429192	-0.322586
		C	0.281104	3.142055	-0.428898
		O	-3.22905	1.261444	0.356806
		C	1.357553	-1.88591	-0.764067

	C	2.605439	-1.39255	-0.373856
	C	2.767798	-0.23523	0.421086
	O	1.811192	0.454702	0.899766
	O	4.006647	0.269896	0.733062
	C	5.181915	-0.40014	0.276025
	H	-1.20437	-2.78419	-0.266755
	H	0.977908	0.773149	-1.230903
	H	-5.26744	-1.95657	0.716604
	H	-4.97703	-0.45198	-0.206985
	H	-4.53714	-0.53378	1.518363
	H	0.055244	4.16906	-0.151104
	H	0.646087	3.115229	-1.459243
	H	1.023107	2.708655	0.243927
	H	-2.92329	2.179936	0.336444
	H	1.291622	-2.88254	-1.184066
	H	3.48323	-1.95704	-0.657031
	H	6.020676	0.189641	0.642982
	H	5.21856	-0.44362	-0.817496
	H	5.250538	-1.41421	0.683066
trans-MS	C	-0.94126	-1.50544	-0.000045
	C	-2.31489	-1.15341	-0.000042
	C	-2.70645	0.22013	-0.000065
	C	-1.68916	1.197821	-0.000013
	C	-0.35645	0.846834	0
	C	0.060654	-0.54462	0.000013
	O	-3.14779	-2.18972	-0.000046
	C	-4.58885	-2.07613	-0.000024
	O	-2.19138	2.464356	-0.000037
	C	-1.27913	3.569521	0.000396
	O	-4.00408	0.590034	-0.000155
	C	1.451053	-0.92011	0.000063
	C	2.519509	-0.02807	-0.000239
	C	3.879029	-0.47802	0.000032
	O	4.279582	-1.6459	0.00071
	O	4.769824	0.584222	-0.00054

		C	6.151301	0.220738	-0.000127
		H	-0.70784	-2.56193	-0.000006
		H	0.39654	1.62021	0.000063
		H	-4.94317	-3.10289	0.000019
		H	-4.92424	-1.54798	-0.891674
		H	-4.92419	-1.54789	0.891588
		H	-1.89902	4.462841	0.000539
		H	-0.65188	3.553827	-0.895132
		H	-0.65214	3.553342	0.896101
		H	-4.02608	1.561865	-0.000137
		H	1.676794	-1.97984	0.000407
		H	2.372575	1.044868	-0.000826
		H	6.707653	1.157454	-0.000693
		H	6.40687	-0.36728	-0.886245
		H	6.406652	-0.36607	0.886857

Table S5. Optimized Cartesian coordinates of the S_0 and S_1 states of the cis-MS and trans-MS in MeOH.

S_0	cis-MS	C	-1.0432	-1.6019	-0.08454
		C	-2.30195	-1.0351	-0.24438
		C	-2.43789	0.356595	-0.24162
		C	-1.29104	1.158389	-0.06249
		C	-0.03692	0.593457	0.089475
		C	0.11009	-0.81031	0.069765
		O	-3.38806	-1.85107	-0.45143
		C	-4.34436	-1.87712	0.624785
		O	-1.5681	2.49609	-0.06558
		C	-0.48269	3.415803	0.090804
		O	-3.65747	0.923078	-0.40857
		C	1.370582	-1.53016	0.194859
		C	2.686394	-1.20109	0.244525
		C	3.339213	0.116016	0.23872
		O	2.818469	1.184274	0.503073
		O	4.665519	0.153675	-0.05579
		C	5.396392	-0.9979	-0.51886

	H	-0.97694	-2.68326	-0.09991
	H	0.834976	1.208174	0.238451
	H	-5.11393	-2.58894	0.329015
	H	-4.79332	-0.89342	0.77788
	H	-3.86911	-2.21623	1.551014
	H	-0.92619	4.408074	0.04881
	H	0.245511	3.300906	-0.71723
	H	0.013695	3.272955	1.055001
	H	-3.54095	1.883977	-0.36326
	H	1.211355	-2.60601	0.22107
	H	3.35823	-2.04741	0.294851
	H	6.369561	-0.6159	-0.82067
	H	4.906008	-1.46236	-1.3765
	H	5.530555	-1.72903	0.281598
trans-MS	C	-0.90532	-1.41212	-0.21436
	C	-2.27281	-1.14915	-0.24119
	C	-2.72391	0.170278	-0.15923
	C	-1.78308	1.217956	-0.03816
	C	-0.4249	0.952734	-0.01748
	C	0.034081	-0.37825	-0.11126
	O	-3.15406	-2.19056	-0.39893
	C	-3.99004	-2.46318	0.741778
	O	-2.3655	2.449416	0.036523
	C	-1.52475	3.602827	0.13988
	O	-4.05189	0.441778	-0.20367
	C	1.44806	-0.72353	-0.09786
	C	2.501095	0.107425	0.007041
	C	3.871279	-0.42453	0.001348
	O	4.191282	-1.59436	-0.09317
	O	4.773953	0.575929	0.118072
	C	6.159323	0.178647	0.125283
	H	-0.58658	-2.44523	-0.28474
	H	0.281086	1.765917	0.067114
	H	-4.59315	-3.32974	0.474186
	H	-4.64217	-1.6163	0.964954

		H	-3.37689	-2.70065	1.61709
		H	-2.19699	4.456655	0.18269
		H	-0.87346	3.690035	-0.73466
		H	-0.92	3.564289	1.050562
		H	-4.15694	1.401524	-0.12344
		H	1.674093	-1.78368	-0.18304
		H	2.401206	1.181524	0.098861
		H	6.727465	1.100623	0.22256
		H	6.415635	-0.33171	-0.80466
		H	6.362282	-0.48558	0.967087
S_1	cis-MS	C	-2.25422	-0.93035	-0.026193
		C	-2.16809	0.479527	0.025093
		C	-0.94672	1.075063	-0.309787
		C	0.169529	0.308659	-0.66402
		C	0.124899	-1.13055	-0.580435
		O	-3.34208	-1.66677	0.222635
		C	-4.62929	-1.10094	0.555589
		O	-0.97549	2.431366	-0.307316
		C	0.26668	3.149443	-0.436293
		O	-3.24081	1.257149	0.34912
		C	1.353038	-1.88519	-0.733748
		C	2.609433	-1.39278	-0.365173
		C	2.787148	-0.23905	0.431718
		O	1.841521	0.446192	0.936137
		O	4.033759	0.262643	0.720817
		C	5.199617	-0.39198	0.214625
		H	-1.20901	-2.78495	-0.263877
		H	0.984526	0.779148	-1.189021
		H	-5.28465	-1.9607	0.670251
		H	-4.98152	-0.45502	-0.247205
		H	-4.56957	-0.53816	1.485859
		H	0.03913	4.177856	-0.165942
		H	0.620303	3.113262	-1.470088
		H	1.018842	2.727533	0.232952
		H	-2.9434	2.178677	0.323095

	H	1.279731	-2.88858	-1.136933
	H	3.481236	-1.95965	-0.661951
	H	6.045438	0.200669	0.559857
	H	5.197887	-0.42138	-0.879534
	H	5.290459	-1.40993	0.606588
trans-MS	C	-0.97733	-1.45811	-0.197428
	C	-2.31579	-1.15073	-0.218834
	C	-2.71821	0.214034	-0.149652
	C	-1.71861	1.230225	-0.027908
	C	-0.37122	0.916302	-0.002973
	C	0.041396	-0.44491	-0.097215
	O	-3.23761	-2.14007	-0.384399
	C	-4.16436	-2.34433	0.705232
	O	-2.25391	2.465385	0.038679
	C	-1.38722	3.608075	0.136261
	O	-4.01553	0.533053	-0.23151
	C	1.416566	-0.83949	-0.079379
	C	2.505326	0.031644	0.000458
	C	3.856257	-0.45402	0.005956
	O	4.216821	-1.63444	-0.05368
	O	4.768683	0.57679	0.089312
	C	6.144575	0.183166	0.102746
	H	-0.69226	-2.50085	-0.262266
	H	0.362885	1.701017	0.090428
	H	-4.77986	-3.19318	0.41379
	H	-4.79401	-1.4674	0.861187
	H	-3.61759	-2.58287	1.62201
	H	-2.0458	4.471634	0.168393
	H	-0.73403	3.664169	-0.737591
	H	-0.7914	3.55489	1.050296
	H	-4.10185	1.499782	-0.175063
	H	1.632663	-1.90005	-0.138723
	H	2.376562	1.105696	0.055804
	H	6.718287	1.106215	0.168595
	H	6.4077	-0.35892	-0.809655

		H	6.362733	-0.45704	0.961982

Table S6. Optimized Cartesian coordinates of the S_0 and S_1 states of the cis-MS and trans-MS in water.

S_0	cis-MS	C	-1.04361	-1.60181	-0.08572
		C	-2.30221	-1.03455	-0.24513
		C	-2.43799	0.35713	-0.2422
		C	-1.29092	1.158822	-0.06367
		C	-0.03689	0.593418	0.087952
		C	0.109876	-0.81041	0.068337
		O	-3.38917	-1.84974	-0.45156
		C	-4.34177	-1.87871	0.628124
		O	-1.56767	2.496419	-0.06681
		C	-0.48234	3.416208	0.09023
		O	-3.6577	0.923419	-0.40848
		C	1.37029	-1.53069	0.192809
		C	2.68614	-1.20186	0.243157
		C	3.339025	0.115135	0.24082
		O	2.818372	1.182684	0.509165
		O	4.664817	0.153974	-0.05475
		C	5.395983	-0.99671	-0.52062
		H	-0.97747	-2.68318	-0.10102
		H	0.835116	1.208024	0.236623
		H	-5.11283	-2.58918	0.333002
		H	-4.78953	-0.89515	0.785757
		H	-3.86334	-2.22084	1.551534
		H	-0.92595	4.408421	0.048831
		H	0.245825	3.301938	-0.71793
		H	0.013895	3.272845	1.054429
		H	-3.54181	1.884392	-0.3625
		H	1.210954	-2.60653	0.217188
		H	3.35792	-2.04831	0.291582
		H	6.369105	-0.613844	-0.821386
		H	4.905504	-1.458916	-1.37935
		H	5.52992	-1.729594	0.278165

	trans-MS	C	-0.90576	-1.41205	-0.21537
		C	-2.27316	-1.14864	-0.24206
		C	-2.72421	0.170707	-0.15945
		C	-1.78327	1.218397	-0.03858
		C	-0.42514	0.952884	-0.01809
		C	0.033728	-0.37821	-0.11211
		O	-3.15534	-2.18956	-0.39974
		C	-3.98631	-2.46542	0.743918
		O	-2.36528	2.449758	0.036513
		C	-1.52412	3.60293	0.140609
		O	-4.05235	0.44192	-0.20325
		C	1.447641	-0.72359	-0.09868
		C	2.500672	0.107356	0.006517
		C	3.870953	-0.42436	0.001107
		O	4.190996	-1.59447	-0.09389
		O	4.773365	0.575656	0.118605
		C	6.159007	0.178879	0.126363
		H	-0.58728	-2.44524	-0.28577
		H	0.280787	1.766111	0.066648
		H	-4.59061	-3.33131	0.476788
		H	-4.63743	-1.61903	0.972235
		H	-3.3694	-2.705	1.616005
		H	-2.19615	4.456894	0.183915
		H	-0.8727	3.690405	-0.73382
		H	-0.91933	3.563554	1.051229
		H	-4.1576	1.401576	-0.12142
		H	1.673318	-1.78381	-0.18405
		H	2.400777	1.181432	0.098553
		H	6.72672	1.101042	0.22416
		H	6.415918	-0.331	-0.80369
		H	6.361759	-0.48538	0.968203
S_1	cis-MS	C	-1.09684	-1.7091	-0.315843
		C	-2.25519	-0.93068	-0.026443
		C	-2.16932	0.479684	0.025168
		C	-0.9475	1.075596	-0.307735

	C	0.169033	0.309506	-0.660294
	C	0.124915	-1.13004	-0.576659
	O	-3.3434	-1.66687	0.220359
	C	-4.63172	-1.10174	0.551248
	O	-0.977	2.431873	-0.30529
	C	0.264426	3.150752	-0.436476
	O	-3.24268	1.256473	0.347117
	C	1.352316	-1.88486	-0.729248
	C	2.610033	-1.39264	-0.364088
	C	2.790187	-0.23975	0.433427
	O	1.846278	0.444388	0.942367
	O	4.037916	0.261383	0.719042
	C	5.202176	-0.39075	0.205022
	H	-1.20959	-2.78502	-0.263497
	H	0.985808	0.780388	-1.182046
	H	-5.2866	-1.96197	0.664742
	H	-4.98282	-0.45613	-0.25223
	H	-4.57374	-0.53906	1.481661
	H	0.036621	4.179094	-0.166194
	H	0.616666	3.114187	-1.4707
	H	1.017891	2.729808	0.232009
	H	-2.94669	2.178508	0.320006
	H	1.277929	-2.88925	-1.129895
	H	3.48087	-1.95971	-0.663356
	H	6.049003	0.202018	0.547489
	H	5.194599	-0.4172	-0.889111
	H	5.296072	-1.40955	0.593881
trans-MS	C	-0.97721	-1.45742	-0.198044
	C	-2.31561	-1.15073	-0.220092
	C	-2.71885	0.213642	-0.1508
	C	-1.71965	1.230611	-0.028227
	C	-0.372	0.917306	-0.003133
	C	0.041379	-0.44338	-0.0977
	O	-3.23771	-2.14017	-0.385503
	C	-4.15898	-2.34795	0.708336

O	-2.25552	2.464881	0.038912
C	-1.3901	3.608726	0.137356
O	-4.01616	0.531343	-0.232275
C	1.416287	-0.83775	-0.080392
C	2.50578	0.032909	0.000548
C	3.856235	-0.45322	0.005809
O	4.216538	-1.63406	-0.054581
O	4.769343	0.576882	0.089772
C	6.145221	0.182896	0.103412
H	-0.69147	-2.50001	-0.262533
H	0.361423	1.702645	0.090415
H	-4.77708	-3.1949	0.416853
H	-4.78676	-1.47087	0.871037
H	-3.60767	-2.59059	1.62127
H	-2.0499	4.471263	0.170436
H	-0.73736	3.666252	-0.736688
H	-0.79406	3.555274	1.051177
H	-4.10421	1.497906	-0.174535
H	1.631958	-1.89836	-0.140792
H	2.377385	1.106941	0.057177
H	6.719121	1.105769	0.169586
H	6.408514	-0.35889	-0.809105
H	6.363095	-0.45736	0.96267

Table S7. Calculated transition properties of cis-SA, trans-SA, cis-MS and trans-MS in three solvents.

		State	λ_{abs}(nm)	Contribution MO	Strength(f)
Cis-SA	Eac	S_1	331.02	(68.250%) H→L	0.4490
		S_2	309.43	(67.349%) H-1→L	0.0861
		S_3	273.35	(67.235%) H-3→L	0.0003
		S_4	243.75	(65.143%) H-2→L	0.0525
		S_5	235.81	(60.964%) H→L+1	0.0701
		S_6	222.41	(60.848%) H-4→L	0.2550
	meoh	S_1	330.58	(67.976%) H→L	0.4291
		S_2	307.46	(67.036%) H-1→L	0.0996
		S_3	273.33	(65.208%) H-3→L	0.0004
		S_4	243.76	(63.288%) H-2→L	0.0413
		S_5	236.06	(60.680%) H→L+1	0.0702

		S_6	222.33	(60.659%) H-4→L	0.2593
	water	S_1	330.66	(67.991%) H→L	0.4291
		S_2	307.16	(67.039%) H-1→L	0.1011
		S_3	273.33	(64.698%) H-3→L	0.0004
		S_4	243.78	(62.855%) H-2→L	0.0393
		S_5	236.12	(60.658%) H→L+1	0.0707
		S_6	222.35	(60.682%) H-4→L	0.2610
trans-SA	Eac	S_1	323.45	(69.557%) H→L	0.6059
		S_2	300.84	(67.746%) H-1→L	0.0459
		S_3	269.84	(52.967%) H-3→L	0.0004
		S_4	242.75	(51.489%) H-2→L	0.0649
		S_5	236.78	(61.857%) H→L+1	0.1095
		S_6	222.56	(61.038%) H-4→L	0.2464
	meoh	S_1	324.68	(69.154%) H→L	0.5781
		S_2	301.50	(67.504%) H-1→L	0.0689
		S_3	267.05	(63.222%) H-3→L	0.0003
		S_4	242.95	(61.416%) H-2→L	0.0545
		S_5	237.08	(61.540%) H→L+1	0.1028
		S_6	222.60	(60.474%) H-4→L	0.2531
	water	S_1	325.04	(69.118%) H→L	0.5764
		S_2	301.58	(67.485%) H-1→L	0.0724
		S_3	266.67	(63.730%) H-3→L	0.0003
		S_4	242.97	(61.951%) H-2→L	0.0529
		S_5	237.16	(61.511%) H→L+1	0.1026
		S_6	222.64	(60.420%) H-4→L	0.2542
Cis-MS	Eac	S_1	328.14	(69.147%) H→L	0.4336
		S_2	303.23	(68.125%) H-1→L	0.0507
		S_3	265.59	(53.069%) H-2→L	0.0021
		S_4	238.91	(56.682%) H→L+1	0.0625
		S_5	237.32	(54.369%) H-3→L	0.0659
		S_6	232.04	(55.796%) H→L+2	0.0229
	meoh	S_1	327.54	(69.041%) H→L	0.4212
		S_2	302.25	(67.991%) H-1→L	0.0545
		S_3	263.31	(50.778%) H-2→L	0.0023
		S_4	238.96	(65.143%) H-2→L	0.0642
		S_5	236.59	(57.939%) H→L+1	0.0638
		S_6	231.03	(53.758%) H-3→L	0.0224
	water	S_1	327.58	(69.048%) H→L	0.4216
		S_2	302.13	(67.990%) H-1→L	0.0549
		S_3	262.97	(50.448%) H-2→L	0.0024
		S_4	238.99	(58.096%) H→L+1	0.0649
		S_5	236.49	(53.608%) H-3→L	0.0640
		S_6	230.91	(54.214 8%) H→L+2	0.0225
trans-MS	Eac	S_1	325.52	(69.763 %) H→L	0.6883

	S_2	299.75	(67.719 %) H-1→L	0.0446
	S_3	263.18	(65.623 %) H-3→L	0.0003
	S_4	240.37	(61.107 %) H-2→L	0.0636
	S_5	239.63	(58.651 %) H→L+1	0.1195
	S_6	231.09	(65.645 %) H→L+2	0.0010
meoh	S_1	326.07	(69.550 %) H→L	0.6660
	S_2	300.99	(67.711 %) H-1→L	0.0596
	S_3	261.13	(66.832 %) H-3→L	0.0003
	S_4	240.30	(55.040 %) H-2→L	0.0720
	S_5	239.28	(50.137 %) H→L+1	0.1061
	S_6	229.52	(65.990 %) H→L+2	0.0010
water	S_1	326.33	(69.532 %) H→L	0.6653
	S_2	301.18	(67.721 %) H-1→L	0.0618
	S_3	260.85	(66.907 %) H-3→L	0.0003
	S_4	240.31	(53.588 %) H-2→L	0.0739
	S_5	239.23	(48.440 %) H→L+1	0.1048
	S_6	229.33	(66.005 %) H→L+2	0.0011

Table S8. Cartesian coordinates of conical intersections of SA in Eac.

C	0.532795	-1.55072	-0.07677783
C	1.785851	-1.05704	0.16762677
C	1.97622	0.32793	0.17641059
C	0.906766	1.175929	-0.08548876
C	-0.34825	0.687137	-0.34314119
C	-0.54574	-0.70924	-0.31925753
O	2.795008	-1.92696	0.48383024
C	3.960042	-2.01434	-0.36619601
O	1.252217	2.504162	-0.06195735
C	0.263253	3.534795	-0.19436443
O	3.216327	0.834396	0.40898579
C	-1.85441	-1.27537	-0.6134107
C	-3.02009	-0.5543	-1.07134387
C	-3.70567	-0.44848	0.09555852
O	-3.09682	-1.02968	1.08783156
O	-4.86174	0.213226	0.27903926
H	0.40319	-2.61503	-0.05389725
H	-1.17374	1.332557	-0.54461731
H	4.594892	-2.75077	0.09523753
H	4.464623	-1.06424	-0.41625597

H	3.671995	-2.34299	-1.3568775
H	0.795913	4.463317	-0.08713246
H	-0.48457	3.446296	0.58117465
H	-0.20275	3.490924	-1.16911243
H	3.216352	1.786847	0.42373232
H	-1.87031	-2.35433	-0.6445806
H	-3.32598	-0.36208	-2.07129352
H	-5.17863	0.129807	1.17379845

Table S9. Cartesian coordinates of conical intersections of SA in MeOH.

C	0.532795	-1.55072	-0.07677783
C	1.785851	-1.05704	0.16762677
C	1.97622	0.32793	0.17641059
C	0.906766	1.175929	-0.08548876
C	-0.34825	0.687137	-0.34314119
C	-0.54574	-0.70924	-0.31925753
O	2.795008	-1.92696	0.48383024
C	3.960042	-2.01434	-0.36619601
O	1.252217	2.504162	-0.06195735
C	0.263253	3.534795	-0.19436443
O	3.216327	0.834396	0.40898579
C	-1.85441	-1.27537	-0.6134107
C	-3.02009	-0.5543	-1.07134387
C	-3.70567	-0.44848	0.09555852
O	-3.09682	-1.02968	1.08783156
O	-4.86174	0.213226	0.27903926
H	0.40319	-2.61503	-0.05389725
H	-1.17374	1.332557	-0.54461731
H	4.594892	-2.75077	0.09523753
H	4.464623	-1.06424	-0.41625597
H	3.671995	-2.34299	-1.3568775
H	0.795913	4.463317	-0.08713246
H	-0.48457	3.446296	0.58117465
H	-0.20275	3.490924	-1.16911243
H	3.216352	1.786847	0.42373232

H	-1.87031	-2.35433	-0.6445806
H	-3.32598	-0.36208	-2.07129352
H	-5.17863	0.129807	1.17379845

Table S10. Cartesian coordinates of conical intersections of SA in water.

C	0.532795	-1.55072	-0.07677783
C	1.785851	-1.05704	0.16762677
C	1.97622	0.32793	0.17641059
C	0.906766	1.175929	-0.08548876
C	-0.34825	0.687137	-0.34314119
C	-0.54574	-0.70924	-0.31925753
O	2.795008	-1.92696	0.48383024
C	3.960042	-2.01434	-0.36619601
O	1.252217	2.504162	-0.06195735
C	0.263253	3.534795	-0.19436443
O	3.216327	0.834396	0.40898579
C	-1.85441	-1.27537	-0.6134107
C	-3.02009	-0.5543	-1.07134387
C	-3.70567	-0.44848	0.09555852
O	-3.09682	-1.02968	1.08783156
O	-4.86174	0.213226	0.27903926
H	0.40319	-2.61503	-0.05389725
H	-1.17374	1.332557	-0.54461731
H	4.594892	-2.75077	0.09523753
H	4.464623	-1.06424	-0.41625597
H	3.671995	-2.34299	-1.3568775
H	0.795913	4.463317	-0.08713246
H	-0.48457	3.446296	0.58117465
H	-0.20275	3.490924	-1.16911243
H	3.216352	1.786847	0.42373232
H	-1.87031	-2.35433	-0.6445806
H	-3.32598	-0.36208	-2.07129352
H	-5.17863	0.129807	1.17379845

Table S11. Cartesian coordinates of conical intersections of MS in Eac.

C	0.944752	-1.52578	-0.12957929	C	0.944752
C	2.148056	-0.99075	0.23889624	C	2.148056
C	2.292415	0.40015	0.235252	C	2.292415
C	1.232828	1.240731	-0.15797604	C	1.232828
C	0.043884	0.714603	-0.54731604	C	0.043884
C	-0.12573	-0.69422	-0.53717732	C	-0.12573
O	3.151105	-1.81007	0.68713684	O	3.151105
C	4.382385	-1.92896	-0.06894856	C	4.382385
O	1.563338	2.572657	-0.09335661	O	1.563338
C	0.570486	3.592808	-0.3205891	C	0.570486
O	3.46195	0.942447	0.59682034	O	3.46195
C	-1.34912	-1.2538	-0.92248592	C	-1.34912
C	-2.52122	-0.52682	-1.4013426	C	-2.52122
C	-3.29555	-0.42712	-0.29769735	C	-3.29555
O	-2.81197	-0.87938	0.8179365	O	-2.81197
O	-4.54699	0.107544	-0.33672209	O	-4.54699
C	-5.3305	0.108987	0.86905646	C	-5.3305
H	0.820789	-2.58919	-0.08909789	H	0.820789
H	-0.79205	1.304896	-0.8558913	H	-0.79205
H	4.957106	-2.67797	0.44633163	H	4.957106
H	4.911768	-0.99095	-0.07781774	H	4.911768
H	4.169175	-2.25448	-1.07876502	H	4.169175
H	1.073656	4.527167	-0.14712112	H	1.073656
H	-0.25203	3.476118	0.37046783	H	-0.25203
H	0.21182	3.547901	-1.33888023	H	0.21182
H	3.44842	1.898599	0.59368272	H	3.44842
H	-1.36013	-2.33757	-0.89035069	H	-1.36013
H	-2.79541	-0.45706	-2.42926363	H	-2.79541
H	-6.26514	0.572211	0.5988738	H	-6.26514
H	-4.83836	0.672778	1.64583463	H	-4.83836
H	-5.49501	-0.89798	1.21979258	H	-5.49501

Table S12. Cartesian coordinates of conical intersections of MS in MeOH.

C	0.944752	-1.52578	-0.12957929	C	0.944752
C	2.148056	-0.99075	0.23889624	C	2.148056

C	2.292415	0.40015	0.235252	C	2.292415
C	1.232828	1.240731	-0.15797604	C	1.232828
C	0.043884	0.714603	-0.54731604	C	0.043884
C	-0.12573	-0.69422	-0.53717732	C	-0.12573
O	3.151105	-1.81007	0.68713684	O	3.151105
C	4.382385	-1.92896	-0.06894856	C	4.382385
O	1.563338	2.572657	-0.09335661	O	1.563338
C	0.570486	3.592808	-0.3205891	C	0.570486
O	3.46195	0.942447	0.59682034	O	3.46195
C	-1.34912	-1.2538	-0.92248592	C	-1.34912
C	-2.52122	-0.52682	-1.4013426	C	-2.52122
C	-3.29555	-0.42712	-0.29769735	C	-3.29555
O	-2.81197	-0.87938	0.8179365	O	-2.81197
O	-4.54699	0.107544	-0.33672209	O	-4.54699
C	-5.3305	0.108987	0.86905646	C	-5.3305
H	0.820789	-2.58919	-0.08909789	H	0.820789
H	-0.79205	1.304896	-0.8558913	H	-0.79205
H	4.957106	-2.67797	0.44633163	H	4.957106
H	4.911768	-0.99095	-0.07781774	H	4.911768
H	4.169175	-2.25448	-1.07876502	H	4.169175
H	1.073656	4.527167	-0.14712112	H	1.073656
H	-0.25203	3.476118	0.37046783	H	-0.25203
H	0.21182	3.547901	-1.33888023	H	0.21182
H	3.44842	1.898599	0.59368272	H	3.44842
H	-1.36013	-2.33757	-0.89035069	H	-1.36013
H	-2.79541	-0.45706	-2.42926363	H	-2.79541
H	-6.26514	0.572211	0.5988738	H	-6.26514
H	-4.83836	0.672778	1.64583463	H	-4.83836
H	-5.49501	-0.89798	1.21979258	H	-5.49501

Table S13. Cartesian coordinates of conical intersections of MS in water.

C	0.944752	-1.52578	-0.12957929	C	0.944752
C	2.148056	-0.99075	0.23889624	C	2.148056
C	2.292415	0.40015	0.235252	C	2.292415
C	1.232828	1.240731	-0.15797604	C	1.232828

C	0.043884	0.714603	-0.54731604	C	0.043884
C	-0.12573	-0.69422	-0.53717732	C	-0.12573
O	3.151105	-1.81007	0.68713684	O	3.151105
C	4.382385	-1.92896	-0.06894856	C	4.382385
O	1.563338	2.572657	-0.09335661	O	1.563338
C	0.570486	3.592808	-0.3205891	C	0.570486
O	3.46195	0.942447	0.59682034	O	3.46195
C	-1.34912	-1.2538	-0.92248592	C	-1.34912
C	-2.52122	-0.52682	-1.4013426	C	-2.52122
C	-3.29555	-0.42712	-0.29769735	C	-3.29555
O	-2.81197	-0.87938	0.8179365	O	-2.81197
O	-4.54699	0.107544	-0.33672209	O	-4.54699
C	-5.3305	0.108987	0.86905646	C	-5.3305
H	0.820789	-2.58919	-0.08909789	H	0.820789
H	-0.79205	1.304896	-0.8558913	H	-0.79205
H	4.957106	-2.67797	0.44633163	H	4.957106
H	4.911768	-0.99095	-0.07781774	H	4.911768
H	4.169175	-2.25448	-1.07876502	H	4.169175
H	1.073656	4.527167	-0.14712112	H	1.073656
H	-0.25203	3.476118	0.37046783	H	-0.25203
H	0.21182	3.547901	-1.33888023	H	0.21182
H	3.44842	1.898599	0.59368272	H	3.44842
H	-1.36013	-2.33757	-0.89035069	H	-1.36013
H	-2.79541	-0.45706	-2.42926363	H	-2.79541
H	-6.26514	0.572211	0.5988738	H	-6.26514
C	0.944752	-1.52578	-0.12957929	C	0.944752
C	2.148056	-0.99075	0.23889624	C	2.148056

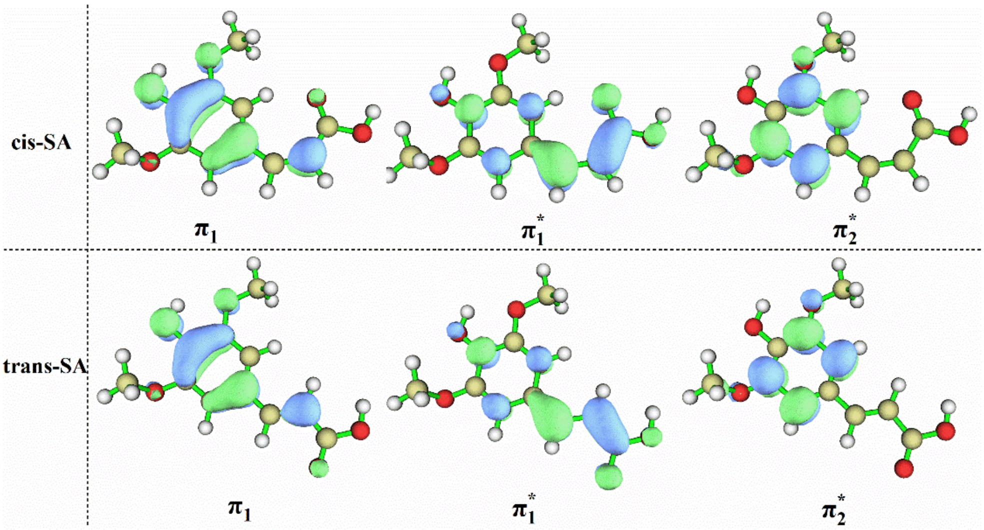

Figure S1. Orbitals used in the active space for CASSCF(2,3) calculations of SA.

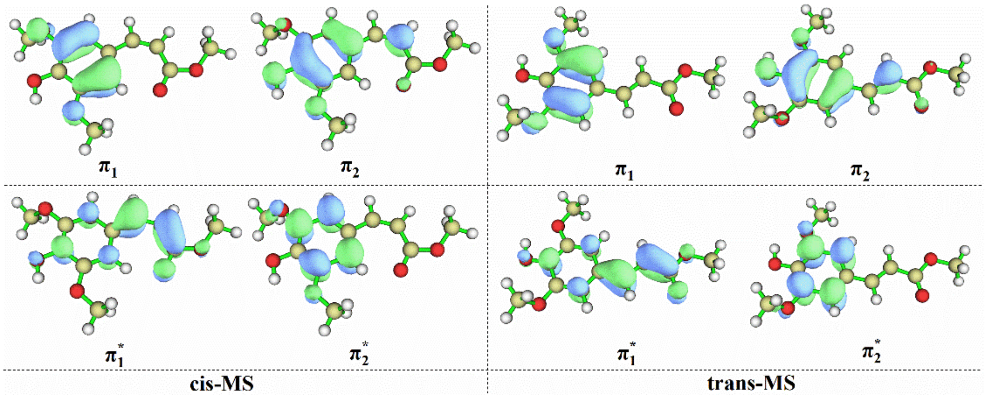

Figure S2. Orbitals used in the active space for CASSCF(4,4) calculations of MS.

As clearly shown in Figure S2, these four orbitals distinctly demonstrate the characteristics of the MS π-conjugated system, encompassing both the C=C double bond region and adjacent aromatic rings. The HOMO-1 and HOMO exhibit typical π-bonding features, while the LUMO and LUMO+1 display pronounced π* antibonding characteristics. Particularly noteworthy is the significant wavefunction distribution of both HOMO and LUMO orbitals in the C=C double bond region - the critical site for photoisomerization. The orbital images reveal appropriate symmetry and shape characteristics, making them well-suited for describing the π→π* transition. For the SA molecule in Figure S1, our natural atomic orbital occupancy analysis revealed an occupation number of merely 0.00006 for the HOMO-1 orbital. Considering both computational complexity and convergence requirements, we rationally selected the HOMO as the representative π orbital. The chosen set of three orbitals unambiguously covers the C=C double bond region, which constitutes the core reactive site for photoisomerization. This selection precisely captures the essential orbitals required to describe the π→π* excitation - the primary pathway for photoisomerization of sinapic acid. The spatial distributions of the selected orbitals are predominantly localized on the reactive molecular moieties, deliberately excluding distal orbitals irrelevant to the isomerization process. The chosen orbital set provides comprehensive coverage of the entire

conjugated system spanning from the C=C double bond to the aromatic rings.

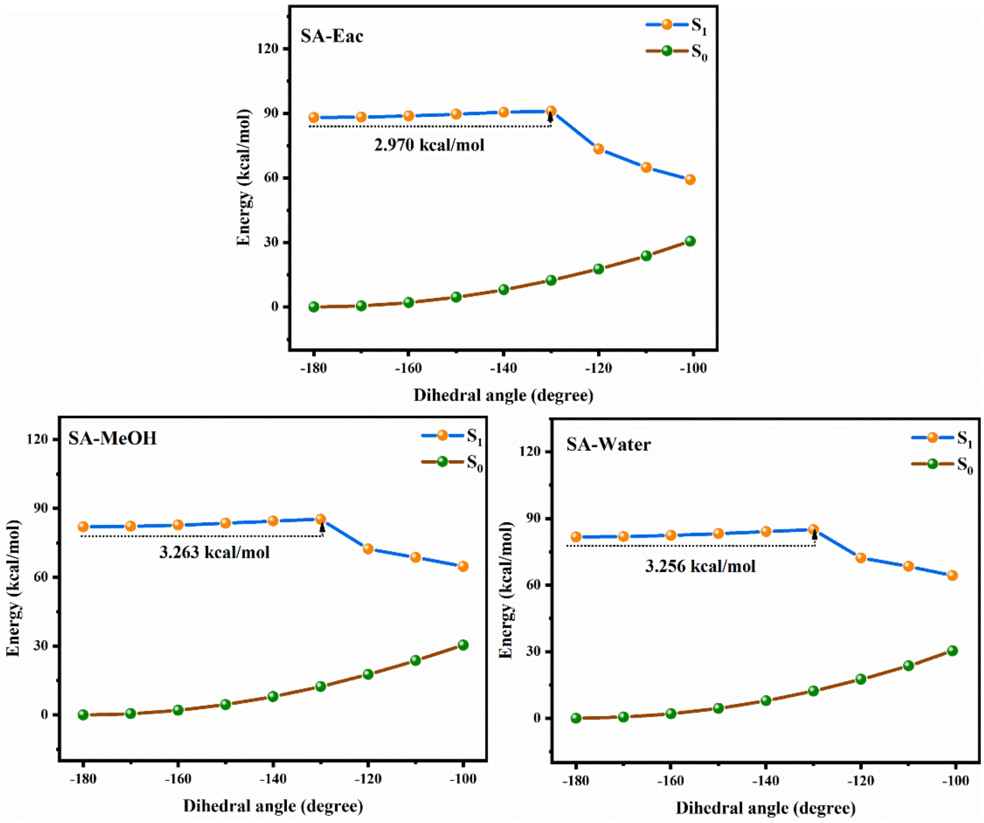

Figure S3. Potential energy surface of SA scanned at the CAM-B3LYP/ 6-311+G(d,p) level.

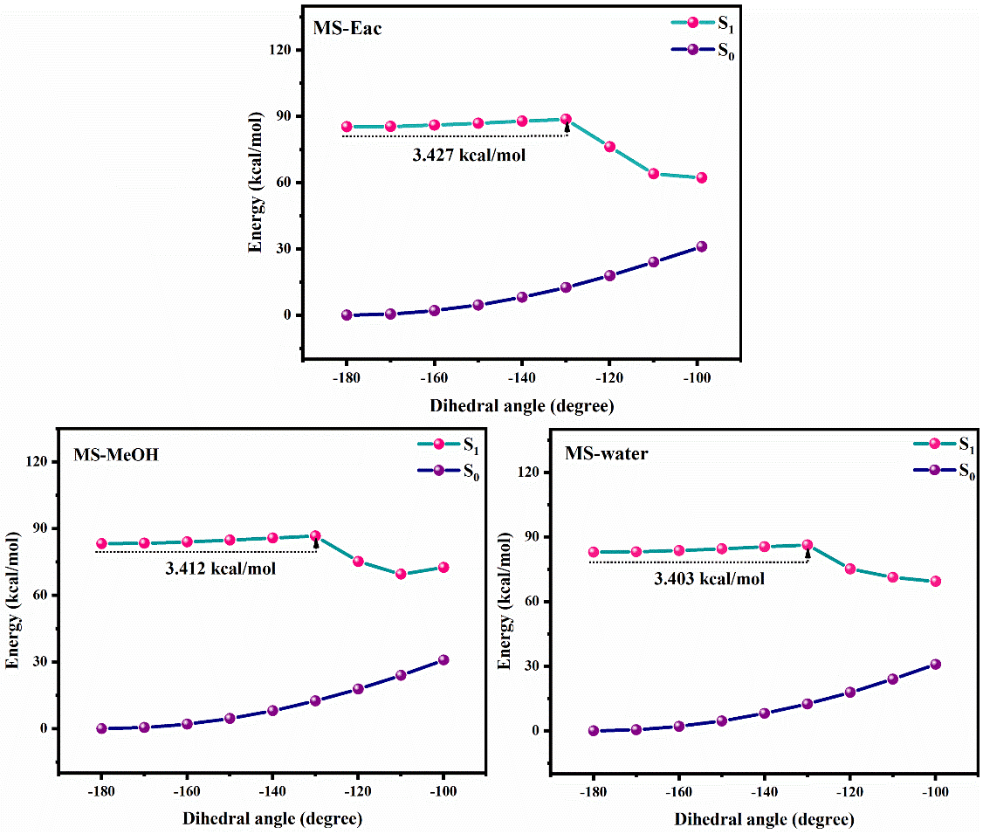

Figure S4. Potential energy surface of MS scanned at the CAM-B3LYP/ 6-311+G(d,p) level.

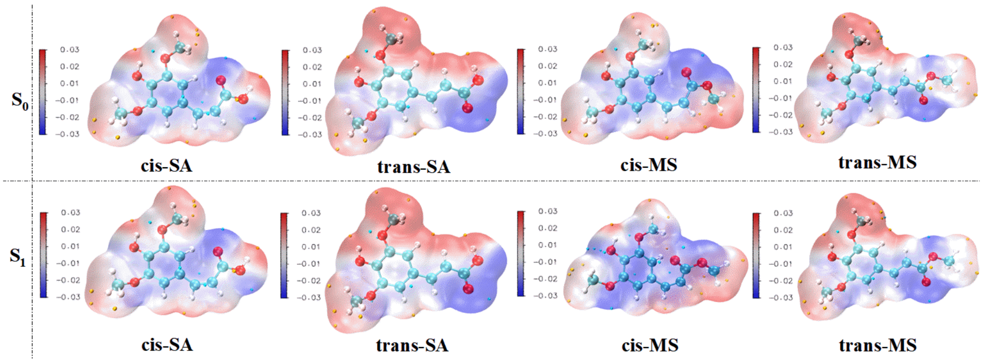

Figure S5. The electrostatic potential distribution of the compounds in MeOH at S_0 and S_1 states.

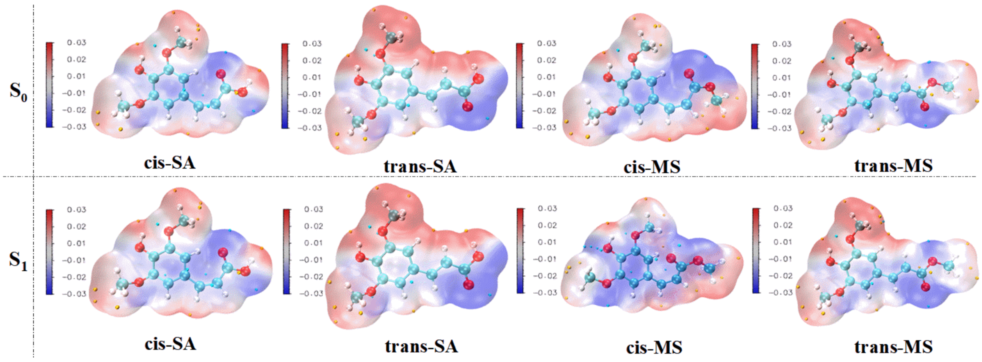

Figure S6. The electrostatic potential distribution of the compounds in Water at S_0 and S_1 states.
